# Chromatin Looping Links Target Genes with Genetic Risk Loci for Dermatological Traits

**DOI:** 10.1016/j.jid.2021.01.015

**Published:** 2021-08

**Authors:** Chenfu Shi, Helen Ray-Jones, James Ding, Kate Duffus, Yao Fu, Vasanthi Priyadarshini Gaddi, Oliver Gough, Jenny Hankinson, Paul Martin, Amanda McGovern, Annie Yarwood, Patrick Gaffney, Steve Eyre, Magnus Rattray, Richard B. Warren, Gisela Orozco

**Affiliations:** 1 Centre for Genetics and Genomics Versus Arthritis, Division of Musculoskeletal and Dermatological Sciences, School of Biological Sciences, Faculty of Biology, Medicine and Health, The University of Manchester, Manchester, United Kingdom; 2 Dermatology Centre, Salford Royal NHS Foundation Trust, NIHR Manchester Biomedical Research Centre, Manchester Academic Health Science Centre, Manchester, United Kingdom; 3 Genes & Human Disease Research Program, Oklahoma Medical Research Foundation, Oklahoma City, Oklahoma, USA; 4 Division of Infection, Immunity and Respiratory Medicine, School of Biological Sciences, University of Manchester, Manchester, United Kingdom; 5 Lydia Becker Institute of Immunology and Inflammation, The University of Manchester, Manchester, United Kingdom; 6 NIHR Manchester Biomedical Research Centre, Manchester University NHS Foundation Trust, Manchester Academic Health Science Centre, Manchester, United Kingdom; 7 Division of Informatics, Imaging and Data Sciences, Faculty of Biology, Medicine and Health, University of Manchester, Manchester, United Kingdom

**Keywords:** AD, atopic dermatitis, eQTL, expression quantitative trait loci, kb, kilobase, KC, keratinocyte, Ps, psoriasis, PsA, psoriatic arthritis, TF, transcription factor

## Abstract

Chromatin looping between regulatory elements and gene promoters presents a potential mechanism whereby disease risk variants affect their target genes. In this study, we use H3K27ac HiChIP, a method for assaying the active chromatin interactome in two cell lines: keratinocytes and skin lymphoma–derived CD8+ T cells. We integrate public datasets for a lymphoblastoid cell line and primary CD4+ T cells and identify gene targets at risk loci for skin-related disorders. Interacting genes enrich for pathways of known importance in each trait, such as cytokine response (psoriatic arthritis and psoriasis) and replicative senescence (melanoma). We show examples of how our analysis can inform changes in the current understanding of multiple psoriasis-associated risk loci. For example, the variant rs10794648, which is generally assigned to *IFNLR1*, was linked to *GRHL3*, a gene essential in skin repair and development, in our dataset. Our findings, therefore, indicate a renewed importance of skin-related factors in the risk of disease.

## Graphical abstract

**Figure:**
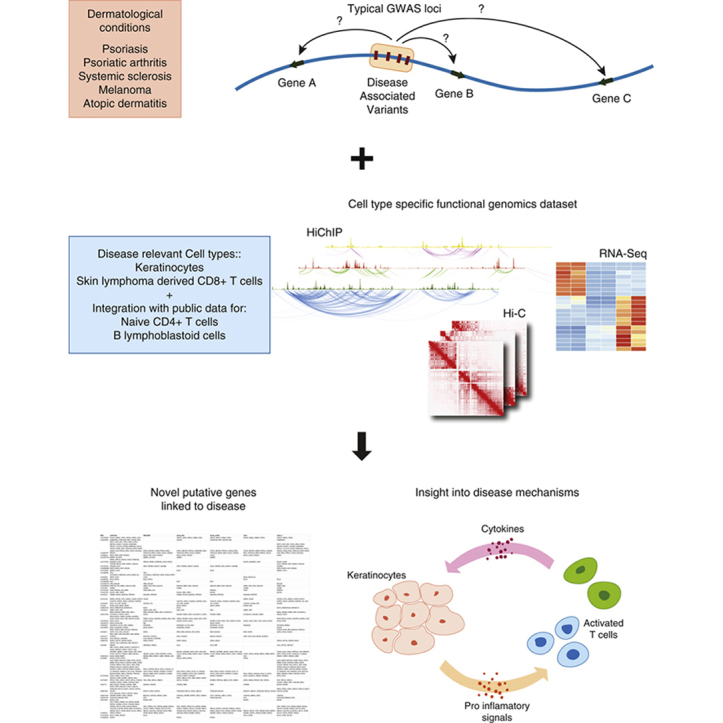

## Introduction

GWASs have uncovered the genetic factors that contribute to disease risk for many complex disorders, including dermatological conditions such as psoriasis (Ps) (Tsoi et al., 2017) or atopic dermatitis (AD) (Paternoster et al., 2015). It is now accepted that the majority of these genetic risk factors do not influence coding sequences directly but rather regulatory regions such as enhancers and promoters, which can be highly cell-type specific (Ernst et al., 2011; Farh et al., 2015; Roadmap Epigenomics Consortium et al., 2015). Many studies have also shown that the effect of these variants is not necessarily mediated by the closest gene because they can affect distal genes through chromatin looping mechanisms ( GTEx Consortium, 2013; GTEx Consortium et al., 2017; Javierre et al., 2016; Nica and Dermitzakis, 2013; Rao et al., 2014; Võsa et al., 2018^1^).

Recently, there has been a growing interest in using chromatin conformation and other functional genomics techniques to investigate how these disease-associated loci lead to disease and identify affected genes. To obtain the resolution necessary to identify enhancer‒promoter interactions, several techniques have been developed that couple Hi-C methods with various enrichment strategies to increase resolution and reduce sequencing cost compared with traditional Hi-C. For example, HiChIP achieves this by using chromatin immunoprecipitation to capture the genomic regions associated with a specific histone modification or protein of interest after Hi-C library preparation (Mumbach et al., 2017, 2016), whereas capture Hi-C uses oligonucleotide baits to capture genomic regions of interest (Dryden et al., 2014). Previous studies have used these techniques to identify causal genes at GWAS loci (Cairns et al., 2016; Dryden et al., 2014; Jäger et al., 2015; Martin et al., 2016, 2015; McGovern et al., 2016; Mumbach et al., 2017), uncovering many important mechanisms and pathways involved in various diseases. These studies have, however, mainly focused on cells derived from blood and immune cells.

Multiple publications have shown that chromatin interactions are cell-type specific and are altered during differentiation and stimulation (Burren et al., 2017; Dixon et al., 2012; Hansen et al., 2018; Mumbach et al., 2017; Rao et al., 2014; Rubin et al., 2017; Schmitt et al., 2016; Siersbæk et al., 2017). Although for many autoimmune diseases associated risk variants primarily affect immune cells, other cell types might also be involved in the disease development (Farh et al., 2015; Mahajan et al., 2018; Mizoguchi et al., 2018). Autoimmune dermatological traits such as Ps, psoriatic arthritis (PsA), and systemic sclerosis are systemic conditions with heterogeneous effects in multiple cell types and are likely to involve a complex interplay between skin-resident cells and immune cells (Albanesi et al., 2018; Mccoy et al., 2017).

Ps is recognized as an immune-mediated condition, and as such, the roles of immune cells in disease have received great attention. However, more recently, there has been a rejuvenated interest in the role of keratinocytes (KCs), which are the most predominant cells in the epidermis and are highly dysregulated in the disease in terms of proliferation and differentiation (Ni and Lai, 2020). KCs respond to T-cell signals by producing proinflammatory cytokines that further contribute to T-cell activation (Albanesi et al., 2018; Benhadou et al., 2018; Lorscheid et al., 2019). A prevalent T-cell‒released signal in inflamed tissue is IFN-γ. In Ps, IFN-γ is released in the classical T helper type 1 pathway (Lowes et al., 2008) and accumulates in psoriatic lesions (Schlaak et al., 1994; Uyemura et al., 1993), promoting epidermal KC apoptosis (Hijnen et al., 2013).

Another recurrent feature in inflammatory skin diseases is the invasion of CD8+ T cells in the inflammation site (Antohe et al. 2019; Cai et al., 2012; Hennino et al., 2007; O’reilly et al., 2012). In Ps, these cells release IL-17, an important factor in disease pathology (Ortega et al., 2009).

In this study, we use HiChIP to map active chromatin interactions genome wide on spontaneously immortalized KCs (HaCaT), unstimulated or stimulated with IFN-γ, and a CD8+ T cell line derived from a cancerous skin plaque (MyLa). In contrast to capture Hi-C, this technique specifically enriches for active regions of the genome. Moreover, it provides interactions genome wide, allowing us to discover candidate genes for a larger set of disorders and to include more recently identified loci in our analysis. We use these new datasets to study disease-associated loci for Ps, PsA, AD, melanoma, and systemic sclerosis and to identify potentially associated genes. Our analysis pipeline is available at https://github.com/ChenfuShi/keratinocyte_gene_link and allows the possibility of using custom input variants (such as non‒genome-wide significant loci or credible SNP sets).

We complement our dataset with matched RNA sequencing and Hi-C. Because the disorders studied have a significant immune component, we include in our analysis publicly available HiChIP data for naïve CD4+ T cells and a B-cell‒like lymphoblastoid cell line (Mumbach et al., 2017) reprocessed with the latest computational tools to investigate the GWAS-associated loci, which might be mediated by these cell populations.

We show that the interacting genes enrich for pathways that are highly relevant to the underlying disease mechanisms. Our results can also inform the association of different genes to specific loci, and we provide examples of this for four distinct Ps loci. Therefore, our findings update our view on the underlying disease mechanisms of these traits, facilitating future studies and drug discovery.

## Results

### A compendium of activity and chromatin interactions in KCs

**Figure 1:**
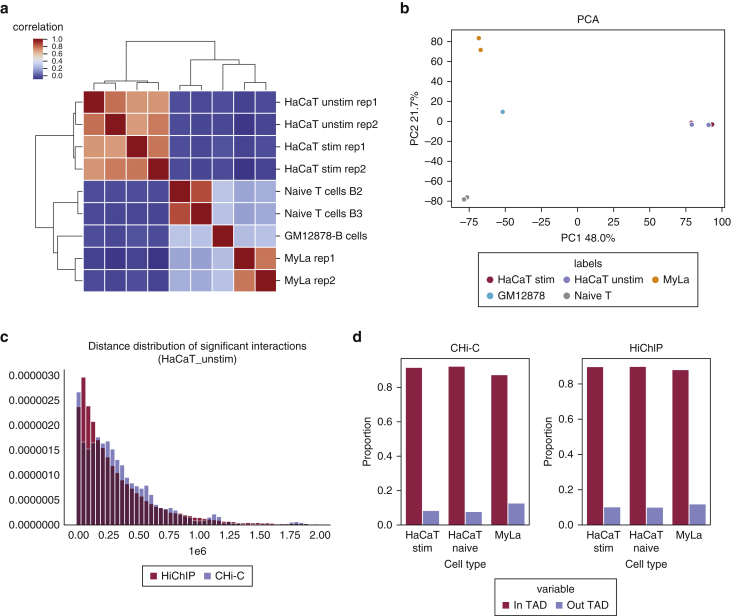
**Properties of the chromatin looping datasets used in this study.** (**a**) Hierarchical clustering of the loops for the individual HiChIP samples using correlation. (**b**) PCA of the loops for the individual HiChIP samples. (**c**) Comparison of the distance distribution of the significant fithichip (HiChIP) and CHiCAGO (CHi-C) interactions for naïve HaCaT HiChIP, a representative example. (d) The proportion of significant interactions that are within TADs and that cross TAD boundaries for region capture Hi-C data and HiChIP data. CHi-C, capture Hi-C; PC, principal component; PCA, principal component analysis; rep, replicate; stim, stimulated; TAD, topologically associating domain; unstim, unstimulated.

Chromatin interactions specific for active regulatory elements, such as enhancers and promoters, were identified using HiChIP targeting the active histone mark H3K27ac. Interactions and peaks are highly cell-type specific, as shown by clustering and principal component analysis (Figure 1a and b and Supplementary Figure S1a and b). We noted some batch effects between replicates that caused an imbalance in the number of detected HiChIP loops between replicates (HaCaT cells) and observed that more variance was explained by batch than by IFN-γ stimulation in HaCaT cells in the principal component analysis plots. Nevertheless, there is substantial overlap in the interaction called between replicates (Supplementary Figure S2 and Supplementary Table S1). As expected, H3K27ac peaks were strongly enriched in GWAS SNPs compared with the background in our data. Moreover, dermatological conditions such as the ones studied in this study possess a stronger enrichment in KCs than other diseases (Supplementary Figure S3).

We identified >50,000 significant interactions from each of our HiChIP datasets (summary statistics in Supplementary Tables S1 and S2). The median interaction distance was 250 kilobase (kb), which is consistent with the results derived from the public datasets (Figure 1c and Supplementary Figure S4a). Moreover, the vast majority (90%) of interactions connected the regions within topologically associating domains identified from our matched Hi-C datasets (Figure 1d). This is consistent with recent reports about topologically associating domains determining the scope of gene regulation (Rao et al., 2014; Rowley and Corces, 2018). We compared this new dataset with our previous study that used capture Hi-C in the same cell lines and targeted GWAS loci from a number of autoimmune disorders (Ray-Jones et al., 2020). We found significant overlap between capture Hi-C and HiChIP, although HiChIP identifies significantly more genes that are specifically active in these cell types (Figure 1c and d, Supplementary Figures S4b and S5a and b, and Supplementary Materials and Methods). Moreover, we recovered most of the interacting genes highlighted in the previous work, such as *IL23A*, *STAT3*, *B3GNT2*, *COMMD1*, *ERRFI1,* and *SOX4* (Ray-Jones et al., 2020).

RNA sequencing for IFN-γ‒stimulated HaCaT cells revealed 535 differentially expressed (447 upregulated and 88 downregulated) genes that were enriched for pathways related to IFN-γ stimulation (Supplementary Figure S5c). This stimulation has a significant impact on the number of interactions that originate from these genes: genes overexpressed on stimulation have 7.08 interactions per gene in stimulated cells compared with 4.48 in unstimulated cells (Supplementary Figure S5d) (×1.58 in these genes compared with ×1.18 overall interactions). Although it seemed that these interactions linked the regions that have increased H3K27 acetylation (mean: ×1.3, median: ×1.64, in stimulated cells compared with that in unstimulated cells), the majority of these linked peaks already have significant levels of H3K27 acetylation before stimulation (Supplementary Figure S5e).

### Chromatin contacts identified by HiChIP pinpoint functionally relevant interactions

We first wanted to test whether HiChIP can successfully discover important features involved in gene regulation. To do this, we used the 535 differentially expressed genes that are activated on IFN-γ stimulation and assumed that these genes are regulated through enhancers binding transcription factors (TFs) related to IFN-γ response. As expected, H3K27ac peaks linked to these genes through HiChIP interactions in HaCaT were enriched for TF motifs that are known to be involved in the response to IFN-γ stimulation and/or viral infection (Ramezani et al., 2018; Schroder et al., 2004) and contained similar motifs to those identified in peaks that had different H3K27ac signal in the stimulated condition (Supplementary Figure S5f). H3K27ac peaks linked through HiChIP interactions to upregulated genes specifically in stimulated cells enriched significantly for *IRF2*, *ISRE,* and *IRF1* motifs compared with the H3K27ac peaks linked to these genes in both conditions (Supplementary Table S3).

Next, we wanted to assess how HiChIP datasets can recapitulate results from large expression quantitative trait loci (eQTL) studies in the discovery of genes dysregulated by GWAS variants. Using Ps loci, we tested our results from naïve T cells and GM12878 cells with the largest blood eQTL database available, eQTLgen (Võsa et al., 2018^1^), as ground truth. Our HiChIP analysis showed a 52% recall rate and 31% specificity compared with that of the genes identified from eQTLs. Using genes that were linked in both HiChIP replicates, we obtained a 46% recall rate and 40% specificity. For comparison, Javierre et al. (2016) reported a recall rate of 25.7% with lead eQTLs.

We then compared our HaCaT KC datasets with the Genotype-Tissue Expression dataset from sun-exposed skin (GTEx Consortium, 2013), resulting in a recall rate of 56% and a specificity of 14.7% (38% and 20%, respectively, with genes reproduced from both replicates). In this study, the Genotype-Tissue Expression dataset contains relatively few eQTLs compared with the eQTLgen database owing to the limited sample size, reducing specificity in our analysis. By comparison, our previous capture Hi-C dataset generated a 21% recall rate and 11% specificity. Interestingly, 38 of 52 eQTLs linked to Ps loci identified in the skin in Genotype-Tissue Expression are recapitulated in the eQTLgen dataset.

These eQTL datasets were generated from slightly different cell populations compared with those analyzed in this study. We have attempted using eQTL datasets generated from pure cell populations (for CD4+ and CD8+ T cells) such as the Kasela et al. (2017) dataset and the Database of Immune Cell Expression eQTL dataset (Schmiedel et al., 2018), but these studies produced very few hits within the studied loci owing to their limited sample size. Regardless, we obtained similar recall rates as reported earlier (22‒66%).

### Using chromatin conformation and functional genomics to dissect disease-associated loci

**Figure 2:**
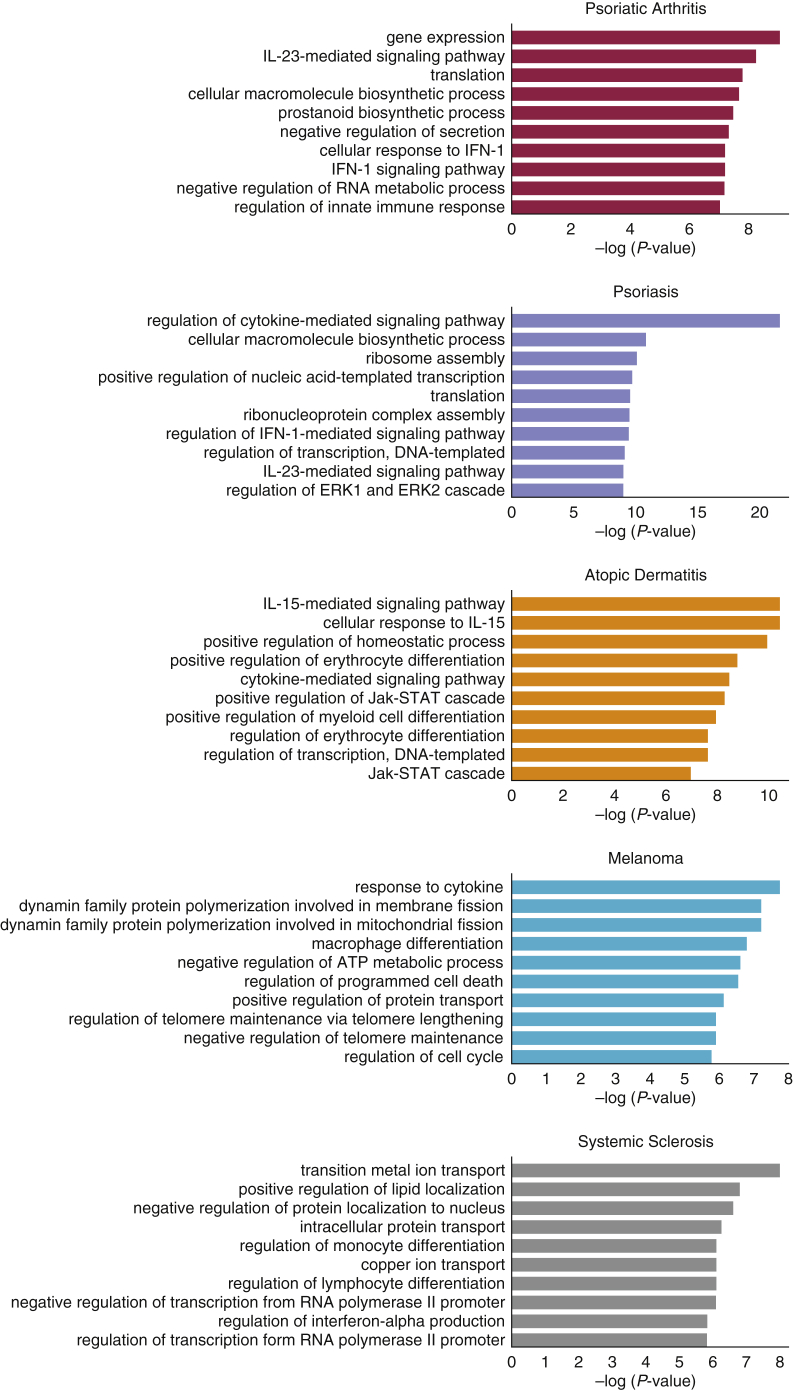
**The top pathways enriched by the genes linked to disease-associated loci from all cell types.** Pathways were identified by EnrichR using genes that were linked in both technical replicates for each condition and/or cell type. ATP, adenosine triphosphate; ERK, extracellular signal–regulated kinase; STAT, signal transducer and activator of transcription.

We next sought to use our dataset to identify the genes whose transcription start sites either overlap or are linked by chromatin interaction to GWAS SNPs associated with PsA, Ps, AD, melanoma, and systemic sclerosis. We identified between 84 and 399 potentially linked genes for each disease studied in our combined datasets (Table 1), with a large fraction of these being recoverable using both replicates for each cell type (Supplementary Figure S6). Importantly, these genes were strongly enriched for disease-relevant pathways. For example, genes linked to melanoma loci were enriched for replicative senescence and cell-cycle pathways, whereas genes linked to Ps and PsA were linked to cytokine signaling and IL-23 (Figure 2), which is a pathway targeted by multiple novel treatments (Sakkas et al., 2019).

**Table 1:** Number of Genes Identified Associated with Each Disease Studied

Disease	Number of Genes Identified
**PsA**	84
**Ps**	399
**Eczema**	154
**Melanoma**	165
**Scleroderma**	182

Abbreviations: Ps, psoriasis; PsA, psoriasis arthritis.

Although some genes were common in all the cell lines (e.g., 12% in Ps), most genes were specific to one or a few cell types (Supplementary Figure S7). Moreover, we found that most loci also implicate multiple genes, with an average of 7.1 genes per loci in Ps. Interestingly, the number of genes implicated by our method correlated significantly with the number of genes that were linked by eQTLs for the same loci (*P* = 4.61e-13) (Supplementary Figure S8). This could be partly caused by the size of the linkage disequilibrium block of the loci, in which the lead SNP implicates a set of coinherited SNPs that can have effects on the expression of many genes.

Using these data, we can begin to provide some functional insight into the mechanisms mediating disease susceptibility. We found that for a significant number of loci, the closest protein-coding genes were not implicated, despite it being possible for linkage disequilibrium blocks to have multiple closest genes. For example, of the 44 Ps loci tested with a linkage disequilibrium block smaller than 100 kb, we linked all the closest genes for only 25 loci. For 14 loci, we did not find any evidence of the closest genes being regulated by the GWAS SNPs in any of the cell lines tested, and for 5 loci, we linked some but not all of the closest genes. Similar numbers can be seen in all the traits studied (Supplementary Table S4). We also identified some loci that do not have any protein-coding genes within 50 kb. We identified 12 of these loci across the diseases studied and provided putative linked genes for all of them in Supplementary File S1.

Looking at a locus, particularly the *ETS1* locus, Ps and AD have distinct associations. However, whereas the Ps locus directly overlaps the promoter of *ETS1*, the association with AD is located 130 kb downstream of the gene. We observed significant chromatin interactions between the AD locus and the *ETS1* promoter in naïve T, Myla, and GM12878 cells (Supplementary Figures S9 and S10). Our data suggest a putative mechanism in which the distinct disease associations at this locus are mediated by a single gene. We follow up with the description of other examples in Ps in the next section.

### HiChIP identifies genes associated with Ps loci in a cell-type‒specific manner

The results from our analysis can be applied to augment the existing understanding of disease-associated loci and to link new genes or change the ones currently linked. In this study, we focus on four Ps-associated loci that show cell-type‒specific and, to our knowledge, previously unreported interactions.

**Figure 3:**
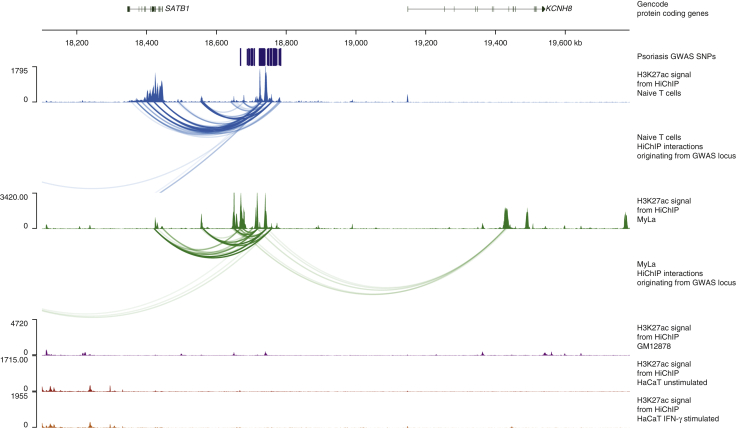
**HiChIP interactions from the *SATB1/KCNH8* locus link *SATB1* as the target gene in this locus.** The tracks (in order) include the following: gencode genes, SNPs associated with psoriasis (r2 > 0.8), H3K27ac signal in naive T cells, significant long-range interactions originating from the psoriasis-associated locus in naïve T cells, H3K27ac signal in MyLa cells, significant long-range interactions originating from the psoriasis associated locus in MyLa cells, H3K27ac signal in GM12878 cells, H3K27ac signal in IFN-γ‒stimulated HaCaT cells, and H3K27ac signal in naive T cells. kb, kilobase.

The Ps locus indexed by SNP rs73178598 is located in an intergenic region overlapping an antisense RNA, *SATB1-AS1*. We found a 240 kb T-cell‒specific interaction present in naïve T and MyLa cells linking this locus with the promoter of *SATB1* in all replicates (Figure 3). Interestingly, for this region, there is an interaction that is present in naïve T cells and Myla but not in KCs or GM12878 with differences visible in Hi-C maps as well (Supplementary Figure S11). Silencing of *SATB1* has been shown to have a similar effect to IFN-γ stimulation on major histocompatibility complex chromatin organization (Kumar et al., 2007) and is an important regulator of regulatory T cells and autoimmunity (Beyer et al., 2011).

The Ps loci indexed by SNP rs9504361 is intronic to *EXOC2* and is typically associated with this gene. However, H3K27ac occupancy at the locus is specific to MyLa cells in our analysis. We detected long-range interactions between this SNP and the promoters of *IRF4* (all replicates) and *DUSP22* (one replicate only), specifically in this cell type (Supplementary Figures S12 and S13). *IRF4* is a lymphocyte-specific TF central to the activation of innate and adaptive immune systems (Huber and Lohoff, 2014), and it was found to be overexpressed in psoriatic skin lesions (Ni et al., 2012). *DUSP22* is a phosphatase that might be involved in the c-Jun N-terminal kinase signaling pathway and has been shown to be associated with lymphomas (Paydas et al., 2019; Zeke et al., 2016).

**Figure 4:**
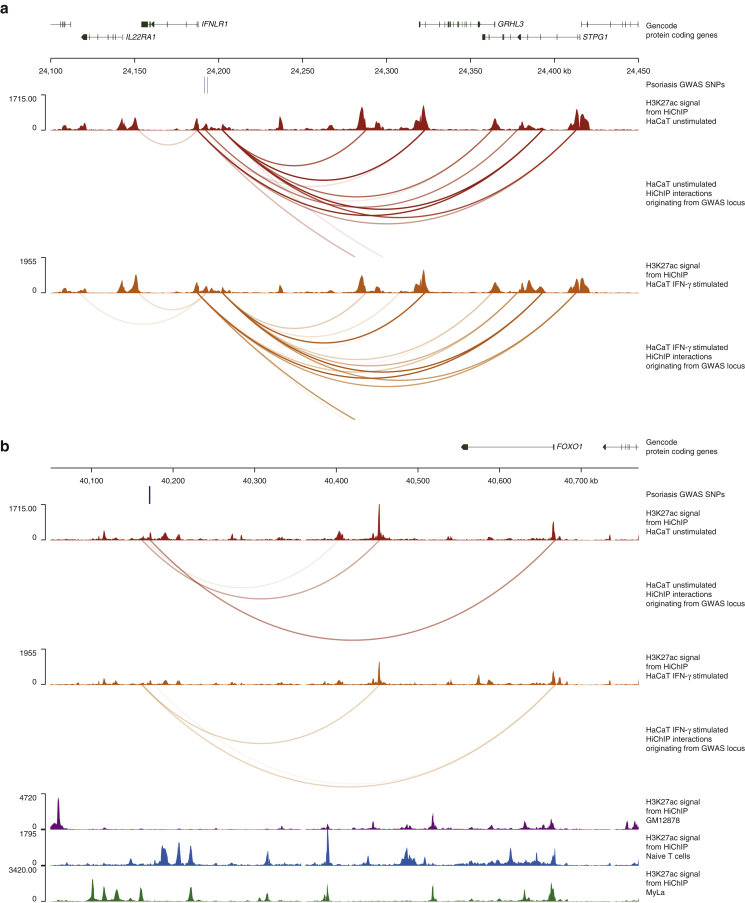
**HiChIP interactions link *GRHL3* and *FOXO1* as candidate genes in psoriasis**. (**a**) HiChIP interactions from the *IFNLR1/GRHL3* locus link *GRHL3* as a candidate gene. The tracks (in order) include the following: gencode genes, SNPs associated with psoriasis (r2 > 0.8), H3K27ac signal in unstimulated HaCaT cells, significant long-range interactions originating from the psoriasis-associated locus in unstimulated HaCaT cells, H3K27ac signal in IFN-γ‒stimulated HaCaT cells, and significant long-range interactions originating from the psoriasis-associated locus in IFN-γ‒stimulated HaCaT cells. (**b**) HiChIP interactions from the *FOXO1* locus link *FOXO1* as a candidate gene. The tracks (in order) include the following: gencode genes, SNPs associated with psoriasis (r2 > 0.8), H3K27ac signal in unstimulated HaCaT cells, significant long-range interactions originating from the psoriasis-associated locus in unstimulated HaCaT cells, H3K27ac signal in IFN-γ‒stimulated HaCaT cells, significant long-range interactions originating from the psoriasis-associated locus in IFN-γ‒stimulated HaCaT cells, H3K27ac signal in GM12878 cells, H3K27ac signal in naïve T cells, and H3K27ac signal in MyLa cells. kb, kilobase.

Another example of a gene target, to our knowledge previously unreported, can be found at the 1p36 locus (rs10794648), which to date has been associated with *IFNLR1* (also known as *IL28-RA*) because it is the closest gene to the associated SNPs (Genetic Analysis of Psoriasis Consortium & the Wellcome Trust Case Control Consortium 2, et al., 2010; Stuart et al., 2015). *IFNLR1* encodes part of a receptor for IFN-γ that is presented in the epidermis and is thought to promote an antiviral response in Ps (Lazear et al., 2015). However, our HiChIP data showed long-range interactions between the Ps SNPs at 1p36 and the distal gene *GRHL3* primarily in HaCaT KCs (all replicates in unstimulated cells and combined only in IFN-γ stimulated cells) (Figure 4a and Supplementary Figures S14 and S15). We further explored the chromatin profile in this region using available chromatin immunoprecipitation sequencing data for enhancer-associated marks (H3K27ac and H3K4me1) and a promoter-associated mark (H3K4me3) in progenitor, differentiating and migrating primary KCs (Klein et al., 2017). We found that *GRHL3* overlaps H3K27ac and H3K4me3 marks in all conditions, indicating that the promoter is active through differentiation (Supplementary Figure S16a). Moreover, we found that the Ps-associated SNPs overlap H3K4me1 in migrating and H3K27ac in migrating and progenitor cells but not H3K4me3 in any condition (Supplementary Figure S16b). These findings suggest that the SNPs overlap an enhancer that is present in migrating and progenitor cells but are active in progenitor cells only. *GRHL3* encodes a TF that stimulates migration. It is upregulated in psoriatic lesions and is required for repair of the epidermal skin barrier after immune-mediated injury (Gordon et al., 2014). Improper regulation of GRHL3 in progenitor cells could adversely affect the migration of KCs, which is known to be highly accelerated in Ps, leading to the formation of the classic skin plaques. Targets of the GRHL3 TF include further GWAS-implicated genes such as *IVL* (involved in KC differentiation) (Watt, 1983) and *KLF4* (TF involved in KC differentiation and skin barrier formation). *KLF4* was recently implicated as the likely functional target gene in the 9q31 locus in our previous study (Ray-Jones et al., 2020).

Finally, the locus indexed by the Ps SNP rs73183592, to our knowledge, previously undescribed mechanism was linked through a long-range interaction spanning about 500 kb to *FOXO1* (Figure 4b and Supplementary Figure S17), a gene with important functions in regulatory T cells. Interestingly, this interaction was identified primarily in KCs in our analysis (all replicates in unstimulated and one replicate only in IFN-γ stimulated) but was only weakly supported in T cells because this enhancer seems to be specific to KCs. Dysregulation of this pathway was also found to be important in the development of Ps (Li et al., 2019; Zhang and Zhang, 2019).

## Discussion

Chromatin conformation and functional genomics studies have the potential to uncover the underlying mechanisms that drive the disease susceptibility of many complex traits. Although these techniques are very promising, there has been a lack of studies in disease-relevant cell types, and as recently evidenced, both chromatin interactions and gene regulation are cell type and stimulation specific (Burren et al., 2017; Dixon et al., 2012; Hansen et al., 2018; Mumbach et al., 2017; Rao et al., 2014; Rubin et al., 2017; Schmitt et al., 2016; Siersbæk et al., 2017).

In this study, we used H3K27ac HiChIP, a modern technique that allows combined analysis of both chromatin conformation and chromatin activity, to create a global study of promoter‒enhancer interactions in KC and CD8+ T-cell lines. Equivalent data have so far only been generated in immune cells with few examples in other cell populations, such as HiChIP in endometrial cancer cells (O’Mara et al., 2019) and promoter capture Hi-C in neuronal cells (Song et al., 2019), cardiomyocytes (Choy et al., 2018), and pancreatic islets (Miguel-Escalada et al., 2019).

After assessing the effectiveness of these techniques in linking functional elements with genes in a cell-type‒specific manner, we show their possible use in studying disease-associated loci. We explore disease-associated SNPs for PsA, Ps, AD, melanoma, and systemic sclerosis and identify all the genes that are linked by chromatin interactions to these variants. We show that these genes are enriched for disease-relevant pathways and provide tables and figures for all the loci (Supplementary File S1 and GitHub repository). We show how these data, using four distinct Ps-associated loci as examples, allow us to identify previously unreported mechanisms and provide functional insight into the diseases studied.

The HiChIP gene targets have the potential to be used as therapeutic targets in drug repurposing and discovery, as recently applied for other diseases (Fang et al., 2019; Martin et al., 2019). As a proof of concept, we tested the genes identified in this work by querying the DrugBank database (Wishart et al., 2008) in search of drugs that are currently used and the ones that could be repurposed. Across the diseases studied, we identified 127 genes that are targeted by approved drugs in some diseases, corresponding to 231 drugs that could be potentially repurposed (Supplementary File S2).

A limitation of this study is that it is based on immortalized cell lines. In the future, these studies should include primary tissues and compare healthy with diseased samples. This has so far been done in a limited way, but early results are showing a significant impact of disease state and genetics on chromatin conformation (Delaneau et al., 2019; Fasolino et al., 2020; Gorkin et al., 2019; Kloetgen et al., 2020). Moreover, although the techniques used in this study are an important strategy to identify potential candidate genes, they require further individual experimental validation (such as genetic perturbation techniques) before they can be used for drug targeting.

In summary, through our analysis, we present a list of potential target genes and pathways for mediating disease risk for five complex diseases, and by documenting individual loci, we highlight some mechanisms by which this risk is mediated. These genes and mechanisms represent a useful resource for further research aimed at characterizing how genetic variation has an impact on disease susceptibility for these and other complex diseases.

## Materials and Methods

For detailed methods, see the Supplementary Materials and Methods.

### Cell culture

HaCaT KC cells (T0020001, Addexbio Technologies, San Diego, CA) were cultured in high-glucose DMEM supplemented with 10% fetal bovine serum and penicillin-streptomycin (final concentration: 100 U penicillin and 0.1 mg/ml streptomycin). For HaCaT stimulation experiments, the media were supplemented with 100 ng/ml recombinant human IFN-γ (285-IF-100; R&D Systems, Minneapolis, MN), and cells were incubated for 8 hours before harvest.

My-La CD8+ cells (95051033, Sigma-Aldrich, St. Loius, MO) were cultured in RPMI 1640 medium supplemented with 10% AB human serum (Sigma Aldrich), 100 U/ml recombinant human IL-2 (Sigma-Aldrich), and penicillin-streptomycin.

### HiChIP experiments

HiChIP libraries were generated according to the Chang Lab protocol (Mumbach et al., 2016). Briefly, 10 million cross-linked cells were lysed, the chromatin was digested with MboI, biotinylated, and ligated. After random shearing, chromatin immunoprecipitation was performed against H3K27ac (ab4729, Abcam, Cambridge, United Kingdom). Decrosslinking and biotin pulldown preceded tagmentation with Tn5 transposase (Illumina, San Diego, CA) at 55 °C for 10 minutes. For more details, see the Supplementary Materials and Methods.

### Linking GWAS loci to putative gene targets

To identify the genes that were linked to disease-associated SNPs, we first identified the transcription start sites of all protein-coding transcripts in the hg38 Gencode V29 annotation (Harrow et al., 2012). We associated all transcripts for which a transcription start site was located within 5 kb of a loop. We also associated the transcripts for which the transcription start site was within 1 kb of an SNP overlapping an H3K27ac peak as identified from HiChIP data. All transcripts were then grouped by gene, and the genes were filtered by an expression level of at least 1 transcript per million in the corresponding cell line.

### Data availability statement

The sequence datasets generated and analyzed during this study are available in the Gene Expression Omnibus repository under accession numbers GSE137906 (for capture Hi-C only) and GSE151193 (for HiChIP, RNA sequencing, and Hi-C with updated processed files).

The code required to reproduce the analysis in this study is available on GitHub. Figures with Hi-C and HiChIP loops for every locus for all the diseases studied in this research are also available in the same repository (https://github.com/ChenfuShi/keratinocyte_gene_link).

## ORCIDs

Chenfu Shi: http://orcid.org/0000-0002-3083-8416

Helen Ray-Jones: http://orcid.org/0000-0002-8884-6865

James Ding: http://orcid.org/0000-0001-7273-9646

Kate Duffus: http://orcid.org/0000-0002-8925-1863

Yao Fu: http://orcid.org/0000-0003-2348-4872

Vasanthi Priyadarshini Gaddi: http://orcid.org/0000-0001-8642-6907

Oliver Gough: http://orcid.org/0000-0002-4737-1023

Jenny Hankinson: http://orcid.org/0000-0002-9021-4031

Paul Martin: http://orcid.org/0000-0002-1016-6851

Amanda McGovern: http://orcid.org/0000-0001-7727-3283

Annie Yarwood: http://orcid.org/0000-0001-5026-9815

Patrick Gaffney: http://orcid.org/0000-0002-1580-869X

Steve Eyre: http://orcid.org/0000-0002-1251-6974

Magnus Rattray: http://orcid.org/0000-0001-8196-5565

Richard B Warren: http://orcid.org/0000-0002-2918-6481

Gisela Orozco: http://orcid.org/0000-0002-3479-0448

## Conflict of Interest

The authors state no conflict of interest.

## Footnotes

1 Võsa U, Claringbould A, Westra H-J, Bonder MJ, Deelen P, Zeng B, et al. Unraveling the polygenic architecture of complex traits using blood eQTL metaanalysis. bioRxiv 2018. doi: https://doi.org/10.1101/447367.

## References

[bib1] Albanesi C., Madonna S., Gisondi P., Girolomoni G. (2018). The interplay between keratinocytes and immune cells in the pathogenesis of psoriasis. Front Immunol.

[bib2] Antohe M., Nedelcu R.I., Nichita L., Popp C.G., Cioplea M., Brinzea A. (2019). Tumor infiltrating lymphocytes: the regulator of melanoma evolution. Oncol Lett.

[bib3] Benhadou F., Mintoff D., Schnebert B., Thio H.B. (2018). Psoriasis and microbiota: a systematic review. Diseases.

[bib4] Beyer M., Thabet Y., Müller R.U., Sadlon T., Classen S., Lahl K. (2011). Repression of the genome organizer SATB1 in regulatory T cells is required for suppressive function and inhibition of effector differentiation. Nat Immunol.

[bib5] Burren O.S., Rubio García A., Javierre B.M., Rainbow D.B., Cairns J., Cooper N.J. (2017). Chromosome contacts in activated T cells identify autoimmune disease candidate genes. Genome Biol.

[bib6] Cai Y., Fleming C., Yan J. (2012). New insights of T cells in the pathogenesis of psoriasis. Cell Mol Immunol.

[bib7] Cairns J., Freire-Pritchett P., Wingett S.W., Várnai C., Dimond A., Plagnol V. (2016). CHiCAGO: robust detection of DNA looping interactions in capture Hi-C data. Genome Biol.

[bib8] Choy M.K., Javierre B.M., Williams S.G., Baross S.L., Liu Y., Wingett S.W. (2018). Promoter interactome of human embryonic stem cell-derived cardiomyocytes connects GWAS regions to cardiac gene networks [published correction appears in Nat Commun 2018:12;9:4792. Nat Commun.

[bib9] Delaneau O., Zazhytska M., Borel C., Giannuzzi G., Rey G., Howald C. (2019). Chromatin three-dimensional interactions mediate genetic effects on gene expression. Science.

[bib10] Dixon J.R., Selvaraj S., Yue F., Kim A., Li Y., Shen Y. (2012). Topological domains in mammalian genomes identified by analysis of chromatin interactions. Nature.

[bib11] Dryden N.H., Broome L.R., Dudbridge F., Johnson N., Orr N., Schoenfelder S. (2014). Unbiased analysis of potential targets of breast cancer susceptibility loci by capture Hi-C. Genome Res.

[bib12] Ernst J., Kheradpour P., Mikkelsen T.S., Shoresh N., Ward L.D., Epstein C.B. (2011). Mapping and analysis of chromatin state dynamics in nine human cell types. Nature.

[bib13] Fang H., De Wolf H., Knezevic B., Burnham K.L., Osgood J., ULTRA-DD Consortium (2019). A genetics-led approach defines the drug target landscape of 30 immune-related traits. Nat Genet.

[bib14] Farh K.K., Marson A., Zhu J., Kleinewietfeld M., Housley W.J., Beik S. (2015). Genetic and epigenetic fine mapping of causal autoimmune disease variants. Nature.

[bib15] Fasolino M., Goldman N., Wang W., Cattau B., Zhou Y., Petrovic J. (2020). Genetic variation in Type 1 diabetes reconfigures the 3D chromatin organization of T cells and alters gene expression. Immunity.

[bib16] Strange A., Capon F., Spencer C.C., Knight J., Weale M.E., Genetic Analysis of Psoriasis Consortium & the Wellcome Trust Case Control Consortium 2 (2010). A genome-wide asociation study identifies new psoriasis susceptibility loci and an interaction betwEn HLA-C and ERAP1. Nat Genet.

[bib17] Gordon W.M., Zeller M.D., Klein R.H., Swindell W.R., Ho H., Espetia F. (2014). A GRHL3-regulated repair pathway suppresses immune-mediated epidermal hyperplasia. J Clin Invest.

[bib18] Gorkin D.U., Qiu Y., Hu M., Fletez-Brant K., Liu T., Schmitt A.D. (2019). Common DNA sequence variation influences 3-dimensional conformation of the human genome. Genome Biol.

[bib19] GTEx Consortium (2013). The Genotype-Tissue Expression (GTEx) project. Nat Genet.

[bib20] GTEx Consortium, Laboratory (2017). Data Analysis &Coordinating Center (LDACC)—Analysis Working Group, Statistical Methods Groups—Analysis Working Group, Enhancing GTEx (eGTEx) Groups, NIH Common Fund, NIH/NCI, et al. Genetic effects on gene expression across human tissues [published correction appears in Nature 2018;553:530]. Nature.

[bib21] Hansen A.S., Cattoglio C., Darzacq X., Tjian R. (2018). Recent evidence that TADs and chromatin loops are dynamic structures. Nucleus.

[bib22] Harrow J., Frankish A., Gonzalez J.M., Tapanari E., Diekhans M., Kokocinski F. (2012). GENCODE: the reference human genome annotation for the ENCODE project. Genome Res.

[bib23] Hennino A., Vocanson M., Toussaint Y., Rodet K., Benetière J., Schmitt A.M. (2007). Skin-infiltrating CD8 + T cells initiate atopic dermatitis lesions. J Immunol.

[bib24] Hijnen D., Knol E.F., Gent Y.Y., Giovannone B., Beijn S.J., Kupper T.S. (2013). CD8 (+) T cells in the lesional skin of atopic dermatitis and psoriasis patients are an important source of IFN-γ, IL-13, IL-17, and IL-22. J Invest Dermatol.

[bib25] Huber M., Lohoff M. (2014). IRF4 at the crossroads of effector T-cell fate decision. Eur J Immunol.

[bib26] Jäger R., Migliorini G., Henrion M., Kandaswamy R., Speedy H.E., Heindl A. (2015). Capture Hi-C identifies the chromatin interactome of colorectal cancer risk loci. Nat Commun.

[bib27] Javierre B.M., Burren O.S., Wilder S.P., Kreuzhuber R., Hill S.M., Sewitz S. (2016). Lineage-specific genome architecture links enhancers and non-coding disease variants to target gene promoters. Cell.

[bib28] Kasela S., Kisand K., Tserel L., Kaleviste E., Remm A., Fischer K. (2017). Pathogenic implications for autoimmune mechanisms derived by comparative eQTL analysis of CD4+ versus CD8+ T cells. PLoS Genet.

[bib29] Klein R.H., Lin Z., Hopkin A.S., Gordon W., Tsoi L.C., Liang Y. (2017). GRHL3 binding and enhancers rearrange as epidermal keratinocytes transition between functional states. PLoS Genet.

[bib30] Kloetgen A., Thandapani P., Ntziachristos P., Ghebrechristos Y., Nomikou S., Lazaris C. (2020). Three-dimensional chromatin landscapes in T cell acute lymphoblastic leukemia. Nat Genet.

[bib31] Kumar P.P., Bischof O., Purbey P.K., Notani D., Urlaub H., Dejean A. (2007). Functional interaction between PML and SATB1 regulates chromatin-loop architecture and transcription of the MHC class I locus. Nat Cell Biol.

[bib32] Lazear H.M., Nice T.J., Diamond M.S. (2015). Interferon-λ: immune functions at barrier surfaces and beyond. Immunity.

[bib33] Li B., Lei J., Yang L., Gao C., Dang E., Cao T. (2019). Dysregulation of Akt-FOXO1 pathway leads to dysfunction of regulatory T cells in patients with psoriasis. J Invest Dermatol.

[bib34] Lorscheid S., Müller A., Löffler J., Resch C., Bucher P., Kurschus F.C. (2019). Keratinocyte-derived IκBζ drives psoriasis and associated systemic inflammation. JCI Insight.

[bib35] Lowes M.A., Kikuchi T., Fuentes-Duculan J., Cardinale I., Zaba L.C., Haider A.S. (2008). Psoriasis vulgaris lesions contain discrete populations of Th1 and Th17 T cells. J Invest Dermatol.

[bib36] Mahajan A., Taliun D., Thurner M., Robertson N.R., Torres J.M., Rayner N.W. (2018). Fine-mapping type 2 diabetes loci to single-variant resolution using high-density imputation and islet-specific epigenome maps. Nat Genet.

[bib37] Martin P., Ding J., Duffus K., Gaddi V.P., McGovern A., Ray-Jones H. (2019). Chromatin interactions reveal novel gene targets for drug repositioning in rheumatic diseases. Ann Rheum Dis.

[bib38] Martin P., McGovern A., Massey J., Schoenfelder S., Duffus K., Yarwood A. (2016). Identifying causal genes at the multiple sclerosis associated region 6q23 using capture Hi-C. PLoS One.

[bib39] Martin P., McGovern A., Orozco G., Duffus K., Yarwood A., Schoenfelder S. (2015). Capture Hi-C reveals novel candidate genes and complex long-range interactions with related autoimmune risk loci. Nat Commun.

[bib40] Mccoy S.S., Reed T.J., Berthier C.C., Tsou P.S., Liu J., Gudjonsson J.E. (2017). Scleroderma keratinocytes promote fibroblast activation independent of transforming growth factor beta. Rheumatology (Oxford).

[bib41] McGovern A., Schoenfelder S., Martin P., Massey J., Duffus K., Plant D. (2016). Capture Hi-C identifies a novel causal gene, IL20RA, in the pan-autoimmune genetic susceptibility region 6q23. Genome Biol.

[bib42] Miguel-Escalada I., Bonàs-Guarch S., Cebola I., Ponsa-Cobas J., Mendieta-Esteban J., Atla G. (2019). Human pancreatic islet three-dimensional chromatin architecture provides insights into the genetics of type 2 diabetes. Nat Genet.

[bib43] Mizoguchi F., Slowikowski K., Wei K., Marshall J.L., Rao D.A., Chang S.K. (2018). Functionally distinct disease-associated fibroblast subsets in rheumatoid arthritis. Nat Commun.

[bib44] Mumbach M.R., Rubin A.J., Flynn R.A., Dai C., Khavari P.A., Greenleaf W.J. (2016). HiChIP: efficient and sensitive analysis of protein-directed genome architecture. Nat Methods.

[bib45] Mumbach M.R., Satpathy A.T., Boyle E.A., Dai C., Gowen B.G., Cho S.W. (2017). Enhancer connectome in primary human cells identifies target genes of disease-associated DNA elements. Nat Genet.

[bib46] Ni A., Chen H., Wu Y., Li W., Chen S., Li J. (2012). Expression of IRF-4 and IBP in skin lesions of patients with psoriasis vulgaris. J Huazhong Univ Sci Technol Med Sci.

[bib47] Ni X., Lai Y. (2020). Keratinocyte: a trigger or an executor of psoriasis?. J Leukoc Biol.

[bib48] Nica A.C., Dermitzakis E.T. (2013). Expression quantitative trait loci: present and future. Philos Trans R Soc Lond B Biol Sci.

[bib49] O’Mara T.A., Spurdle A.B., Glubb D.M., Endometrial Cancer Association Consortium (2019). Analysis of promoter-associated chromatin interactions reveals biologically relevant candidate target genes at endometrial cancer risk loci. Cancers (Basel).

[bib50] O’reilly S., Hügle T., Van Laar J.M. (2012). T cells in systemic sclerosis: a reappraisal. Rheumatology (Oxford).

[bib51] Ortega C., Fernández-AS, Carrillo J.M., Romero P., Molina I.J., Moreno J.C. (2009). IL-17-producing CD8 + T lymphocytes from psoriasis skin plaques are cytotoxic effector cells that secrete Th17-related cytokines. J Leukoc Biol.

[bib52] Paternoster L., Standl M., Waage J., Baurecht H., Hotze M., Strachan D.P. (2015). Multi-ancestry genome-wide association study of 21,000 cases and 95,000 controls identifies new risk loci for atopic dermatitis. Nat Genet.

[bib53] Paydas S., Bagir E.K., Ergin M., Seydaoglu G., Boz A. (2019). The role of DUSP22 (dual specificity phosphatase 22) gene expression in the prognosis of low grade lymphomas. Hematol Oncol.

[bib54] Ramezani A., Nahad M.P., Faghihloo E. (2018). The role of Nrf2 transcription factor in viral infection. J Cell Biochem.

[bib55] Rao S.S., Huntley M.H., Durand N.C., Stamenova E.K., Bochkov I.D., Robinson J.T. (2014). A 3D map of the human genome at kilobase resolution reveals principles of chromatin looping. Cell.

[bib56] Ray-Jones H., Duffus K., McGovern A., Martin P., Shi C., Hankinson J. (2020). Mapping DNA interaction landscapes in psoriasis susceptibility loci highlights KLF4 as a target gene in 9q31. BMC Biol.

[bib57] Kundaje A., Meuleman W., Ernst J., Bilenky M., Yen A., Roadmap Epigenomics Consortium (2015). Integrative analysis of 111 reference human epigenomes. Nature.

[bib58] Rowley M.J., Corces V.G. (2018). Organizational principles of 3D genome architecture. Nat Rev Genet.

[bib59] Rubin A.J., Barajas B.C., Furlan-Magaril M., Lopez-Pajares V., Mumbach M.R., Howard I. (2017). Lineage-specific dynamic and pre-established enhancer–promoter contacts cooperate in terminal differentiation. Nat Genet.

[bib60] Sakkas L.I., Zafiriou E., Bogdanos D.P. (2019). Mini review: new treatments in psoriatic arthritis. Focus on the IL-23/17 axis. Front Pharmacol.

[bib61] Schlaak J.F., Buslau M., Jochum W., Hermann E., Girndt M., Gallati H. (1994). T cells involved in psoriasis vulgaris belong to the Th1 subset. J Invest Dermatol.

[bib62] Schmiedel B.J., Singh D., Madrigal A., Valdovino-Gonzalez A.G., White B.M., Zapardiel-Gonzalo J. (2018). Impact of genetic polymorphisms on human immune cell gene expression. Cell.

[bib63] Schmitt A.D., Hu M., Jung I., Xu Z., Qiu Y., Tan C.L. (2016). A compendium of chromatin contact maps reveals spatially active regions in the human genome. Cell Rep.

[bib64] Schroder K., Hertzog P.J., Ravasi T., Hume D.A. (2004). Interferon-gamma: an overview of signals, mechanisms and functions. J Leukoc Biol.

[bib65] Siersbæk R., Madsen J.G.S., Javierre B.M., Nielsen R., Bagge E.K., Cairns J. (2017). Dynamic rewiring of promoter-anchored chromatin loops during adipocyte differentiation. Mol Cell.

[bib66] Song M., Yang X., Ren X., Maliskova L., Li B., Jones I.R. (2019). Mapping cis-regulatory chromatin contacts in neural cells links neuropsychiatric disorder risk variants to target genes. Nat Genet.

[bib67] Stuart P.E., Nair R.P., Tsoi L.C., Tejasvi T., Das S., Kang H.M. (2015). Genome-wide association analysis of psoriatic arthritis and cutaneous psoriasis reveals differences in their genetic architecture. Am J Hum Genet.

[bib68] Tsoi L.C., Stuart P.E., Tian C., Gudjonsson J.E., Das S., Zawistowski M. (2017). Large scale meta-analysis characterizes genetic architecture for common psoriasis associated variants. Nat Commun.

[bib69] Uyemura K., Yamamura M., Fivenson D.F., Modlin R.L., Nickoloff B.J. (1993). The cytokine network in lesional and lesion-free psoriatic skin is characterized by a T-helper type 1 cell-mediated response. J Invest Dermatol.

[bib70] Watt F.M. (1983). Involucrin and other markers of keratinocyte terminal differentiation. J Invest Dermatol.

[bib71] Wishart D.S., Knox C., Guo A.C., Cheng D., Shrivastava S., Tzur D. (2008). DrugBank: a KnowledgeBase for drugs, drug actions and drug targets. Nucleic Acids Res.

[bib72] Zeke A., Misheva M., Reményi A., Bogoyevitch M.A. (2016). JNK signaling: regulation and functions based on complex protein-protein partnerships. Microbiol Mol Biol Rev.

[bib73] Zhang M., Zhang X. (2019). The role of PI3K/AKT/FOXO signaling in psoriasis. Arch Dermatol Res.

