# Supplementary material for: Chromatin Looping Links Target Genes with Genetic Risk Loci for Dermatological Traits
Source: J Invest Dermatol. 2021 Aug;141(8):1975–84. doi: 10.1016/j.jid.2021.01.015 (PMC8315765; doi:10.1016/j.jid.2021.01.015)
Supplement: Supplementary tables figures and methods [file mmc1.pdf]

Supplementary Tables, figures and methods for

Chromatin looping links target genes with genetic risk for dermatological traits

Table S1. Summary statistics of the HiChIP datasets used in this project.

Cell Type (HiChIP)	Reads (millions)	Valid interaction pairs (millions)	Number of significant loops
**HaCaT unstim combined (Keratinocytes)**	569.5	273.1	58268
**HaCaT unstim rep 1**	252.2	133.1	13950
**HaCaT unstim rep 2**	317.3	140.1	38118
**HaCaT combined IFN-γ stimulated**	624.8	309.8	68887
**HaCaT IFN-γ stimulated rep 1**	262.9	136.7	14456
**HaCaT IFN-γ stimulated rep 2**	361.9	173.2	47100
**MyLa combined (CD8+ T cells)**	730.2	288.6	51274
**MyLa rep 1**	446.4	190.3	33257
**MyLa rep 2**	283.7	98.3	13743
**CD4+ Naïve T cells combined**	407.5	230.4	54642
**CD4+ Naïve T cells rep 1 (B2)**	190.5	107.1	29352
**CD4+ Naïve T cells rep 2 (B3)**	217.0	123.4	28405
**GM12878 (B-cell)**	504.7	241.3	76451

Table S2. Summary statistics of the Hi-C datasets used in this project.

Cell Type (Hi-C)	Reads (millions)	Valid interaction pairs (millions)
**HaCaT unstim (Keratinocytes)**	169.7	106.2
**HaCaT IFN-γ stimulated**	182.2	121.4
**MyLa (CD8+ T cells)**	190.7	130.0
**Naïve T cell**	773.1	506.7
**GM12878**	1204.8	855.4

Table S3. Significant motifs enriched in peaks linked specifically in stimulated cells.

Motif	q-value
**IRF2**	0.0059
**ISRE**	0.0610
**IRF1**	0.0985

Table S4. Style of loci tested.

Disease	Linked all closest genes	Linked some closest gene	Not linked any closest genes	Tested loci	Loci with no genes nearby
Psoriatic Arthritis	**4**	**0**	**3**	**7**	**0**
Psoriasis	**25**	**5**	**14**	**44**	**4**
Atopic dermatitis	**7**	**3**	**6**	**16**	**4**
Melanoma	**13**	**1**	**7**	**21**	**3**
Systemic sclerosis	**8**	**7**	**3**	**18**	**1**

Figure S1. Properties of the ChIP peaks identified from the HiChIP dataset. A) hierarchical clustering of the HiChIP peaks for the individual samples using correlation distance. B) PCA of the HiChIP peaks for the individual HiChIP samples.

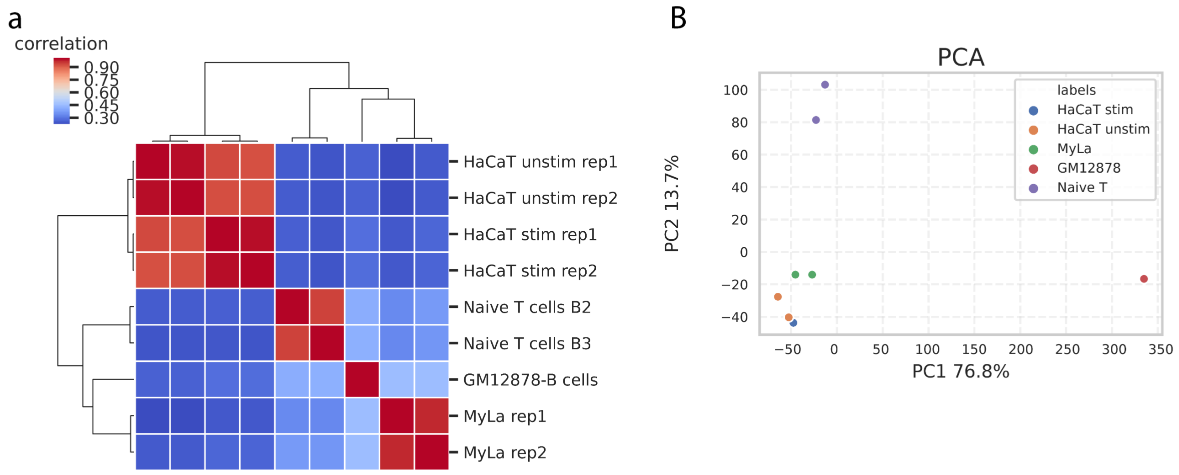

Figure S2. Overlap of loops identified in the two replicates for each condition compared to the loops identified in the merged datasets.

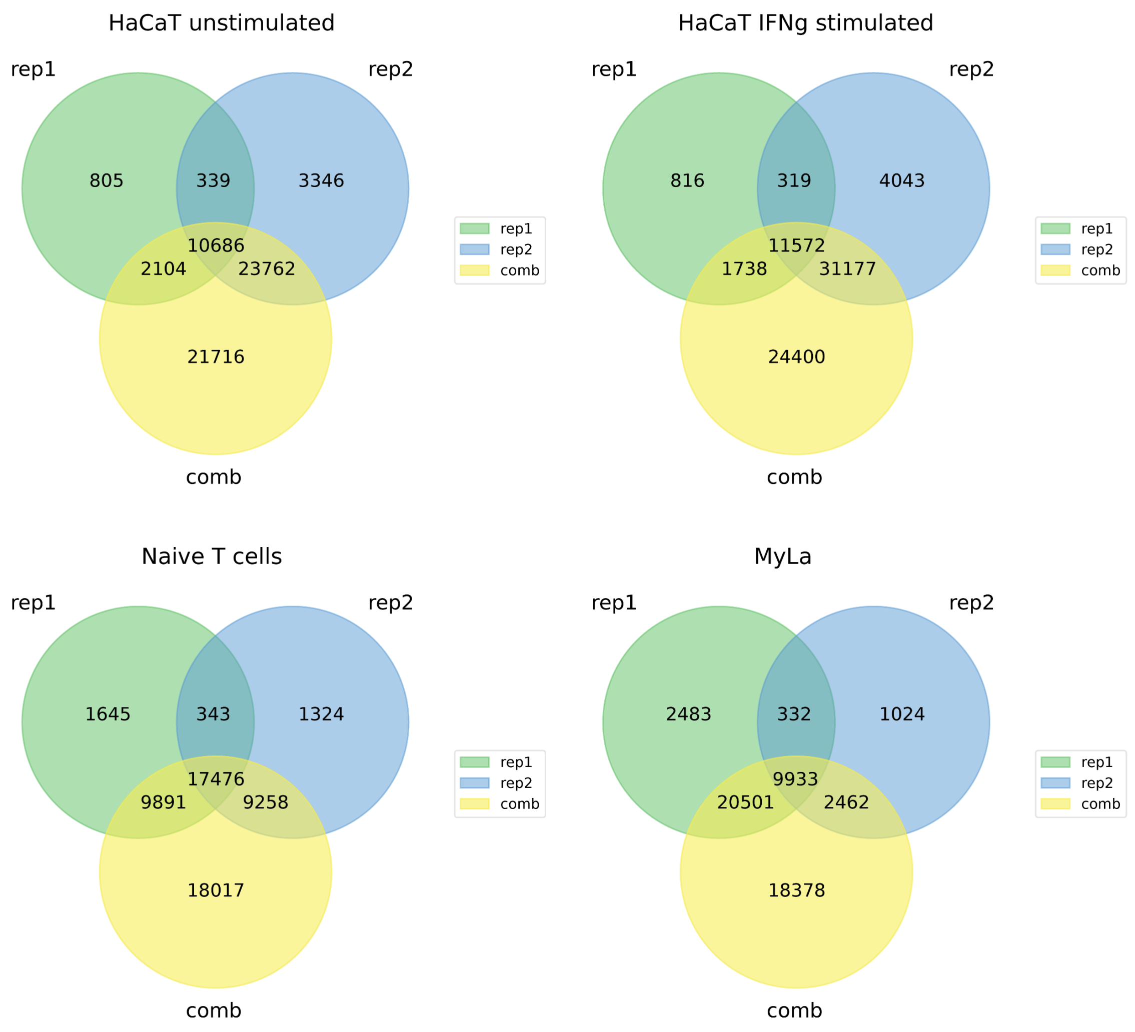

Figure S3. Enrichment over background that disease associated variants have in HiChIP H3K27ac signal. Numbers inside each square represent the number of disease associated variants that directly overlap HiChIP H3K27ac peaks in each of the studied cell types.

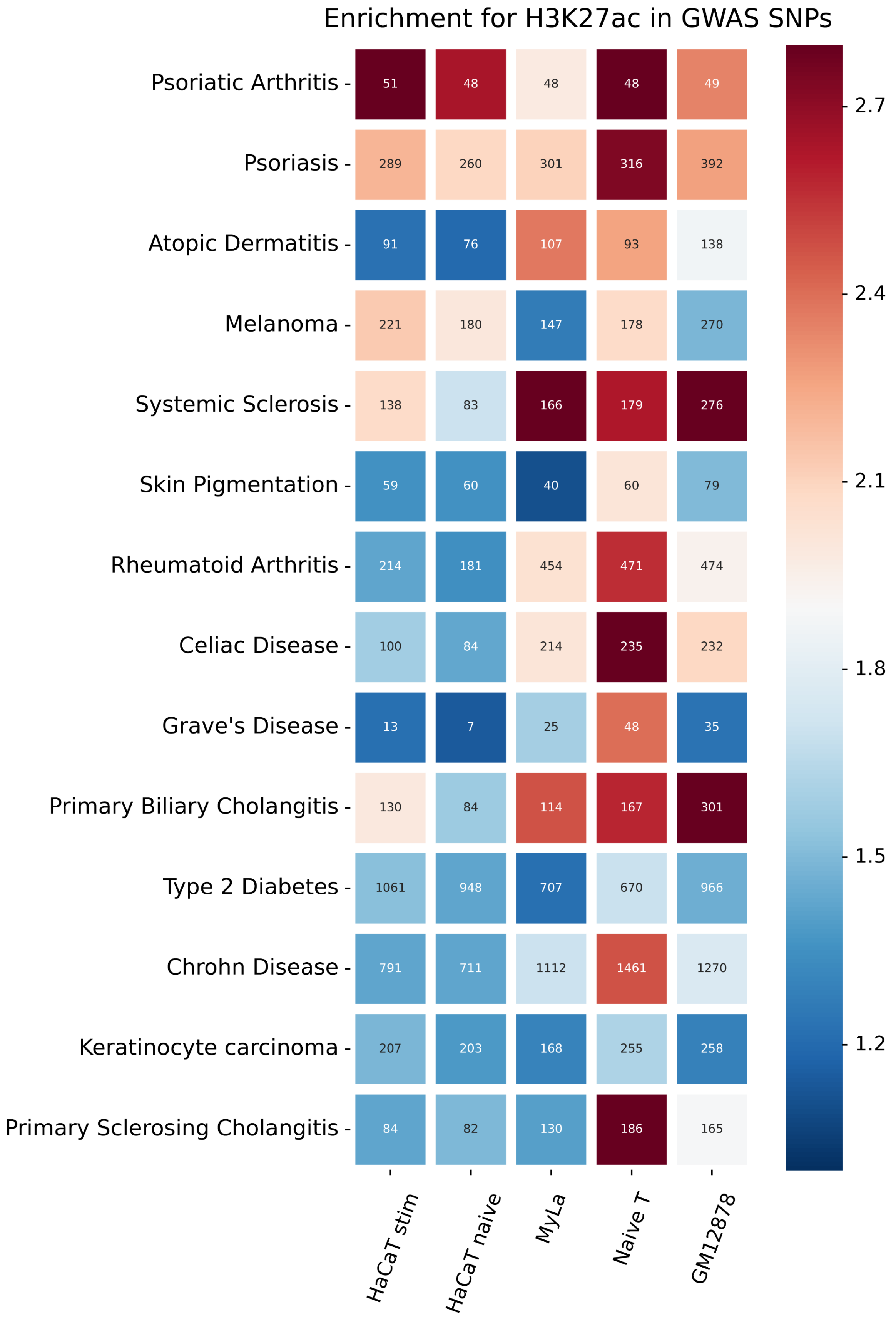

Figure S4. Distribution plots of significant interactions. X-axis: genomic distance (bp); Y-axis: number of interactions. A) HiChIP; B) Capture-Hi-C.

a

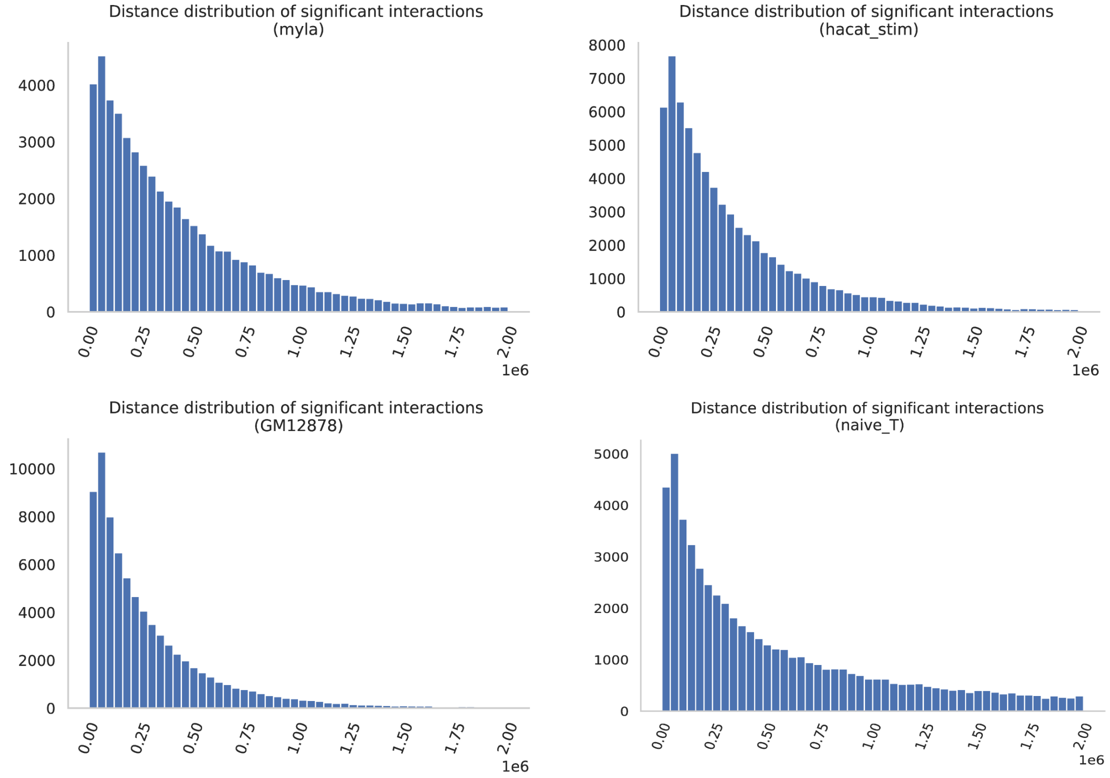

b

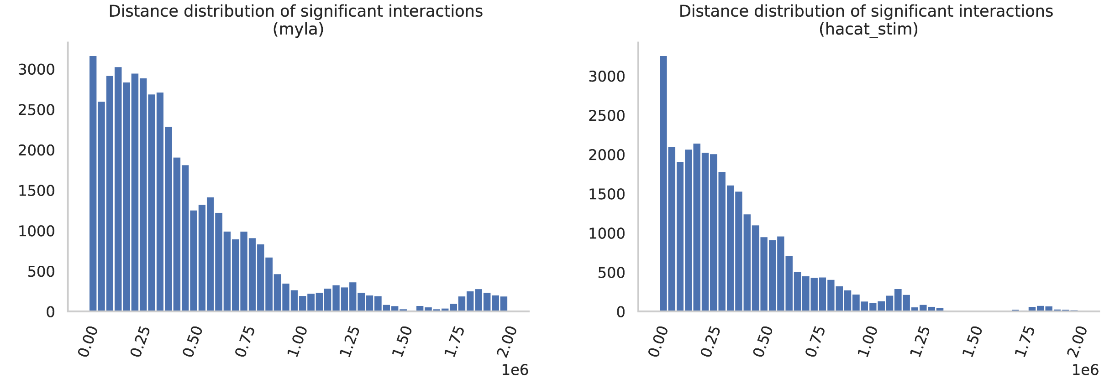

Fibure S5. Functional properties of HiChIP interactions in IFN-γ stimulated HaCaT cells. A) Overlap of the genes identified through region capture Hi-C and HiChIP in HaCaT and MyLa cells for the same regions across all diseases studied. B) Enrichment of H3K27ac peaks in significant Capture-Hi-C interactions vs randomized. Red line indicates 95% confidence interval. C) Pathways identified by DE genes in IFN-γ stim. D) Boxplot displaying the number of interactions that originate from each UP regulated promoter in unstimulated vs IFN -γ stimulated conditions. E) Normalized H3K27ac signal present in the peaks that were linked two UP regulated promoters only in IFN-γ stimulated cells. Although the H3K27ac signal was higher in IFN-γ stimulated cells we find that there were already significant levels of H3K27ac in unstimulated cells. F) Pathways identified from differentially bound HiChIP H3K27ac peaks and HiChIP H3K27ac peaks linked to genes by HiChIP interactions to promoters of genes that were found to be differentially expressed during IFN-γ stimulation.

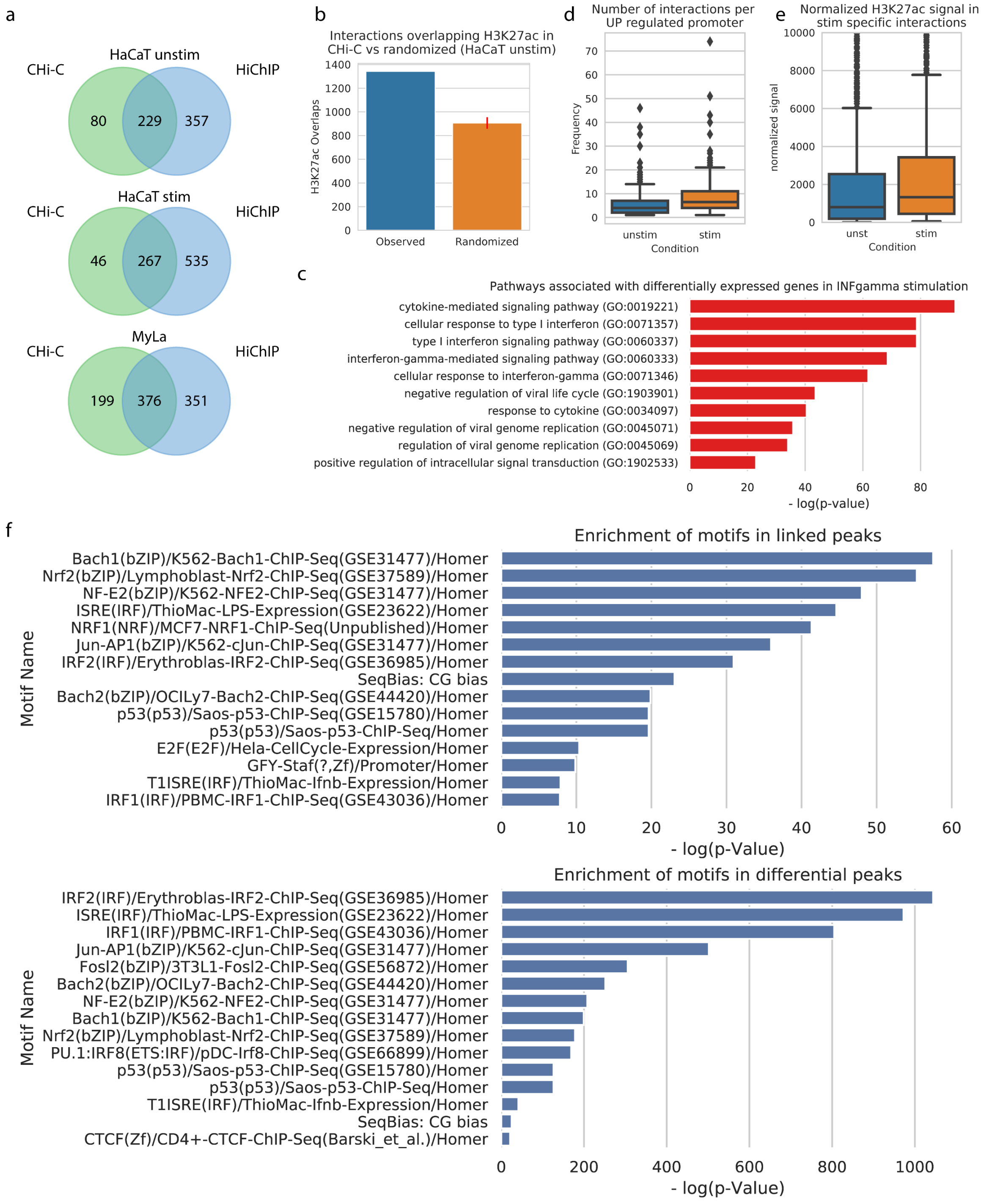
a
HaCaT unstim
CHi-C
HiChIP
80
229
357
HaCaT stim
CHi-C
HiChIP
46
267
535
MyLa
CHi-C
HiChIP
199
376
351
b
Interactions overlapping H3K27ac in CHi-C vs randomized (HaCaT unstim)
H3K27ac Overlaps
Observed
Randomized
d
Number of interactions per UP regulated promoter
Frequency
unstim
stim
Condition
e
Normalized H3K27ac signal in stim specific interactions
normalized signal
unst
stim
Condition
c
Pathways associated with differentially expressed genes in INFgamma stimulation
cytokine-mediated signaling pathway (GO:0019221)
cellular response to type I interferon (GO:0071357)
type I interferon signaling pathway (GO:0060337)
interferon-gamma-mediated signaling pathway (GO:0060333)
cellular response to interferon-gamma (GO:0071346)
negative regulation of viral life cycle (GO:1903901)
response to cytokine (GO:0034097)
negative regulation of viral genome replication (GO:0045071)
regulation of viral genome replication (GO:0045069)
positive regulation of intracellular signal transduction (GO:1902533)
- log(p-value)
f
Enrichment of motifs in linked peaks
Motif Name
Bach1(bZIP)/K562-Bach1-ChIP-Seq(GSE31477)/Homer
Nrf2(bZIP)/Lymphoblast-Nrf2-ChIP-Seq(GSE37589)/Homer
NF-E2(bZIP)/K562-NFE2-ChIP-Seq(GSE31477)/Homer
ISRE(IRF)/ThioMac-LPS-Expression(GSE23622)/Homer
NRF1(NRF)/MCF7-NRF1-ChIP-Seq(Unpublished)/Homer
Jun-AP1(bZIP)/K562-cJun-ChIP-Seq(GSE31477)/Homer
IRF2(IRF)/Erythroblas-IRF2-ChIP-Seq(GSE36985)/Homer
SeqBias: CG bias
Bach2(bZIP)/OCILy7-Bach2-ChIP-Seq(GSE44420)/Homer
p53(p53)/Saos-p53-ChIP-Seq(GSE15780)/Homer
p53(p53)/Saos-p53-ChIP-Seq/Homer
E2F(E2F)/Hela-CellCycle-Expression/Homer
GFY-Staf(?,Zf)/Promoter/Homer
T1ISRE(IRF)/ThioMac-Ifnb-Expression/Homer
IRF1(IRF)/PBMC-IRF1-ChIP-Seq(GSE43036)/Homer
- log(p-Value)
Enrichment of motifs in differential peaks
Motif Name
IRF2(IRF)/Erythroblas-IRF2-ChIP-Seq(GSE36985)/Homer
ISRE(IRF)/ThioMac-LPS-Expression(GSE23622)/Homer
IRF1(IRF)/PBMC-IRF1-ChIP-Seq(GSE43036)/Homer
Jun-AP1(bZIP)/K562-cJun-ChIP-Seq(GSE31477)/Homer
Fosl2(bZIP)/3T3L1-Fosl2-ChIP-Seq(GSE56872)/Homer
Bach2(bZIP)/OCILy7-Bach2-ChIP-Seq(GSE44420)/Homer
NF-E2(bZIP)/K562-NFE2-ChIP-Seq(GSE31477)/Homer
Bach1(bZIP)/K562-Bach1-ChIP-Seq(GSE31477)/Homer
Nrf2(bZIP)/Lymphoblast-Nrf2-ChIP-Seq(GSE37589)/Homer
PU.1:IRF8(ETS:IRF)/pDC-Irf8-ChIP-Seq(GSE66899)/Homer
p53(p53)/Saos-p53-ChIP-Seq(GSE15780)/Homer
p53(p53)/Saos-p53-ChIP-Seq/Homer
T1ISRE(IRF)/ThioMac-Ifnb-Expression/Homer
SeqBias: CG bias
CTCF(Zf)/CD4+-CTCF-ChIP-Seq(Barski_et_al.)/Homer
- log(p-Value)

Figure S6. Venn diagram of genes identified in each cell type in each replicate for psoriasis.

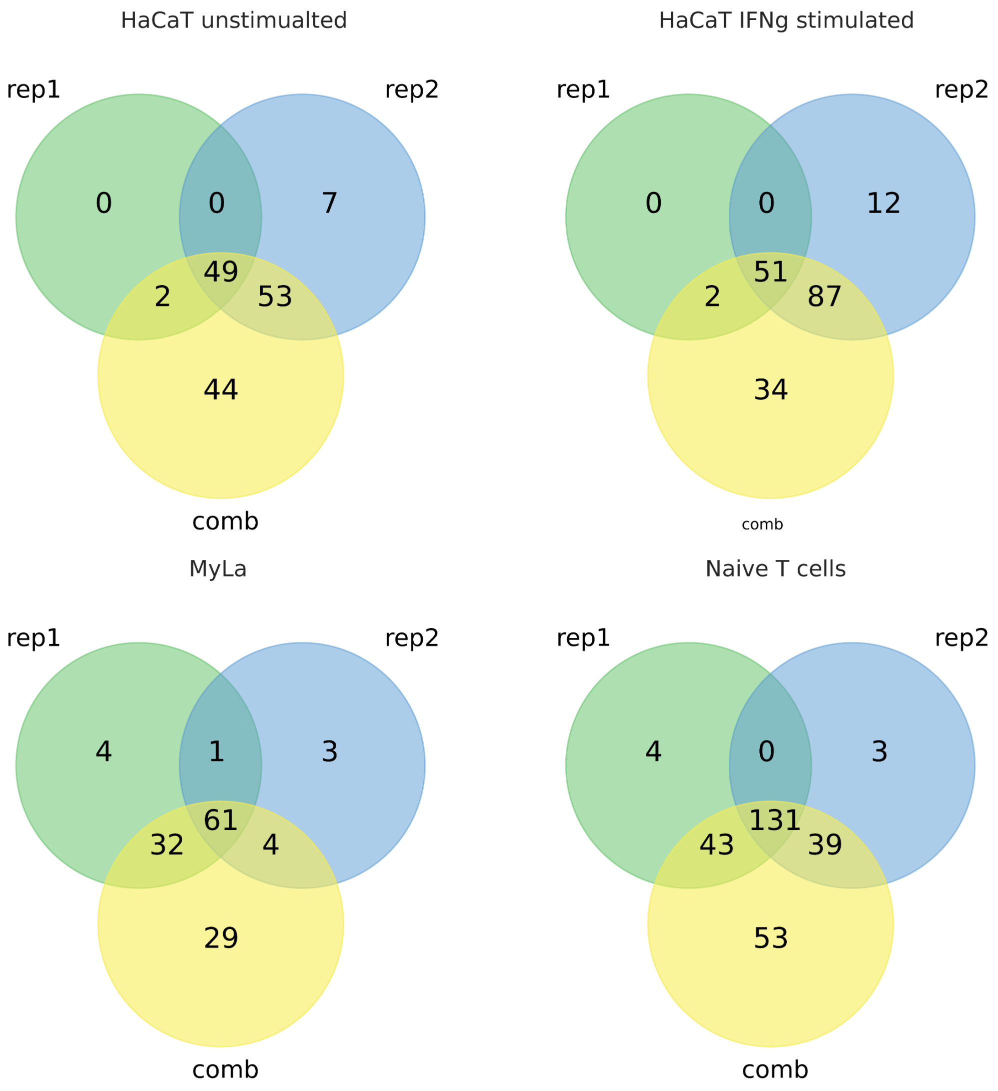

Figure S7. Venn diagram representing the number of genes identified by cell type for all diseases.

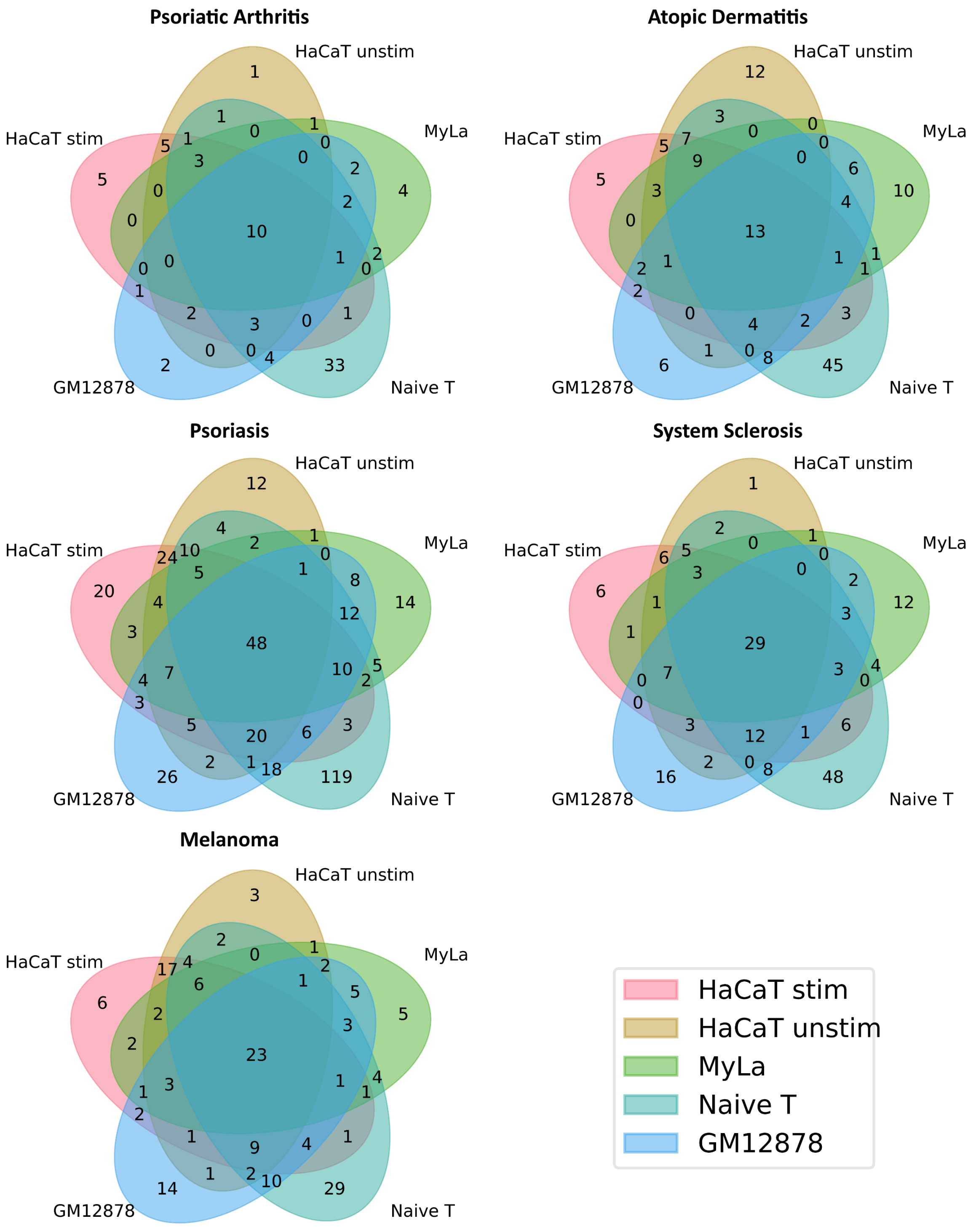

Figure S8. Correlation between number of genes identified by HiChIP vs eQTL. The number of genes linked by HiChIP in Naïve T cells and GM12878 cells correlates with the number of genes linked by eQTL from eQTLgen (R^2 = 0.604).

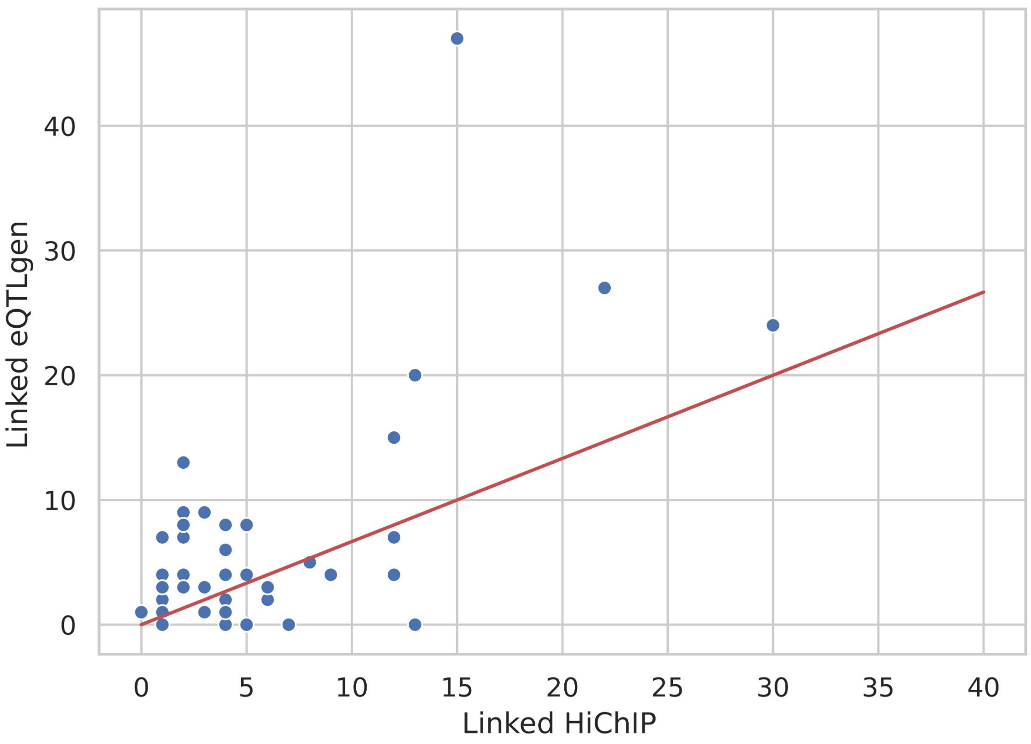

Figure S9. HiChIP interactions from the *ETS1* locus provide a functional mechanism for this locus in atopic dermatitis. Tracks (in order): Gencode genes; SNPs associated with Atopic Dermititis ($r^2 > 0.8$); SNPs associated with Psoriasis ($r^2 > 0.8$); H3K27ac signal in naïve T cells; Significant long range interactions originating from the atopic dermatitis associated locus in naïve T cells; H3K27ac signal in GM12878 cells; Significant long range interactions originating from the atopic dermatitis associated locus in GM12878 cells; H3K27ac signal in MyLa cells; Significant long range interactions originating from the atopic dermatitis associated locus in MyLa cells; H3K27ac signal in unstimulated HaCaT cells; H3K27ac signal in IFN-γ stimulated HaCaT cells.

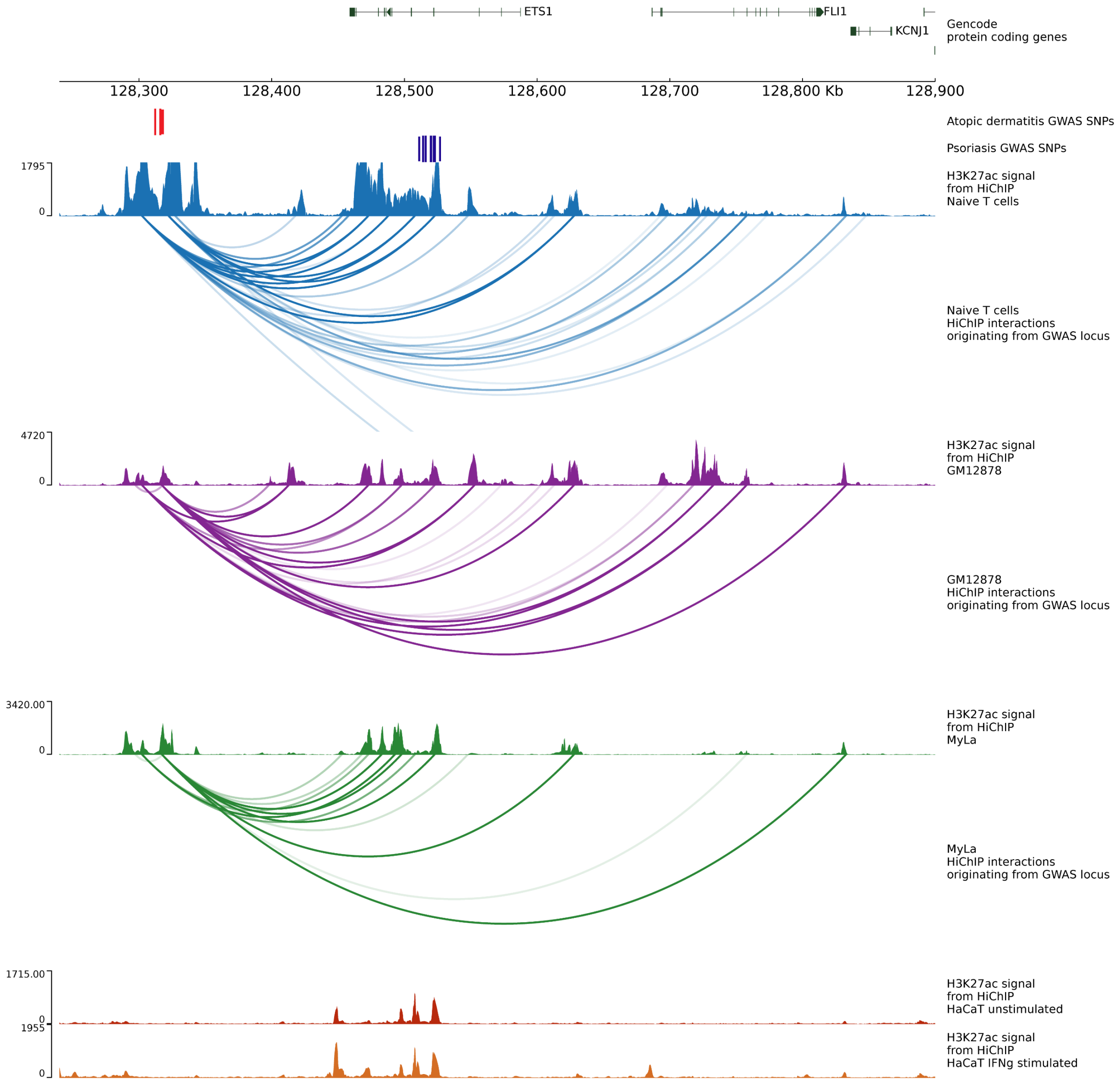

Figure S10. Hi-C contact maps for the ETS1 locus.

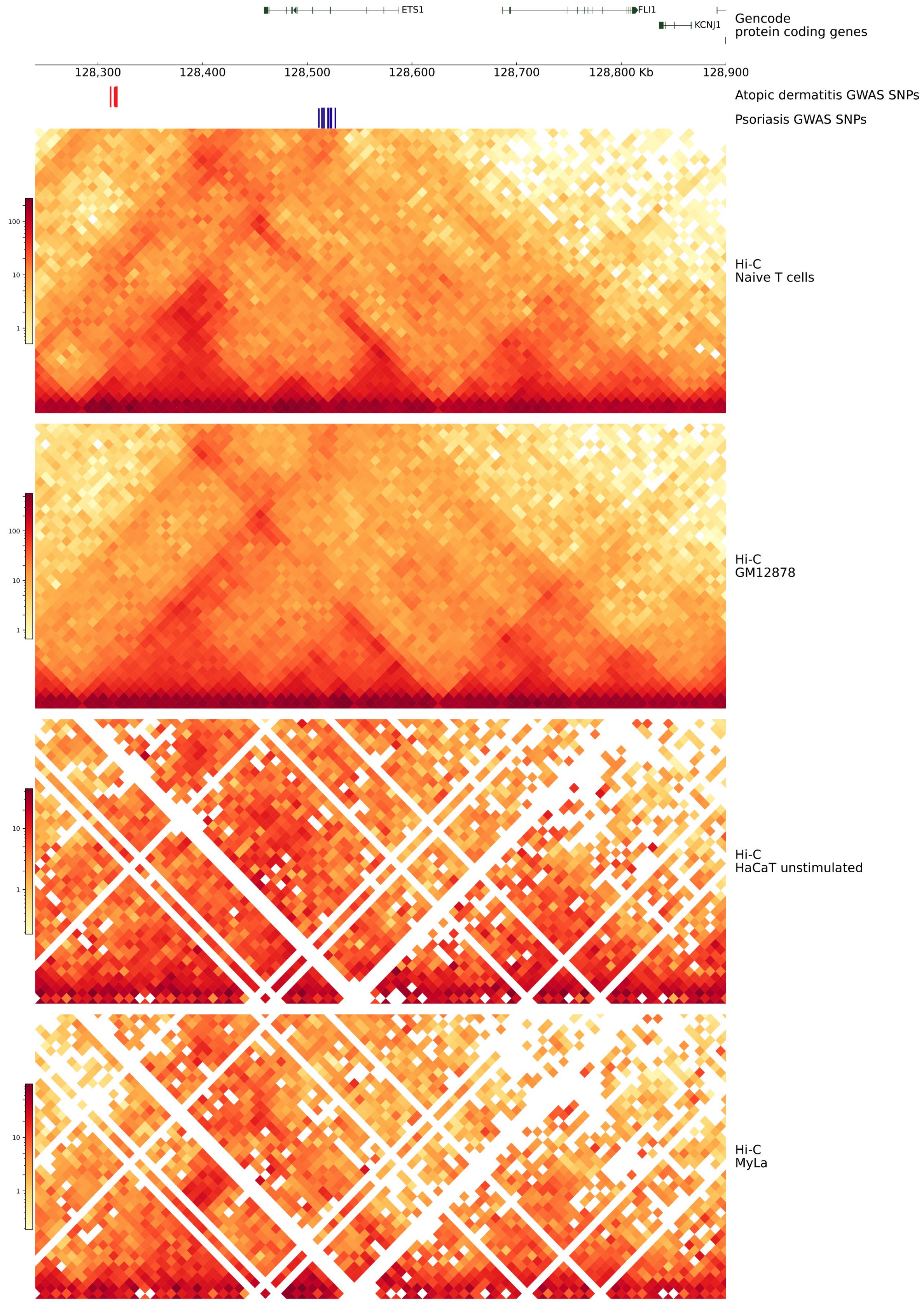

Figure S11. Hi-C contact maps for the SATB1 locus. Hi-C contact maps show a cell type specific loop (highlighted in green) that is present between the psoriasis SNPs and the *SATB1* promoter.

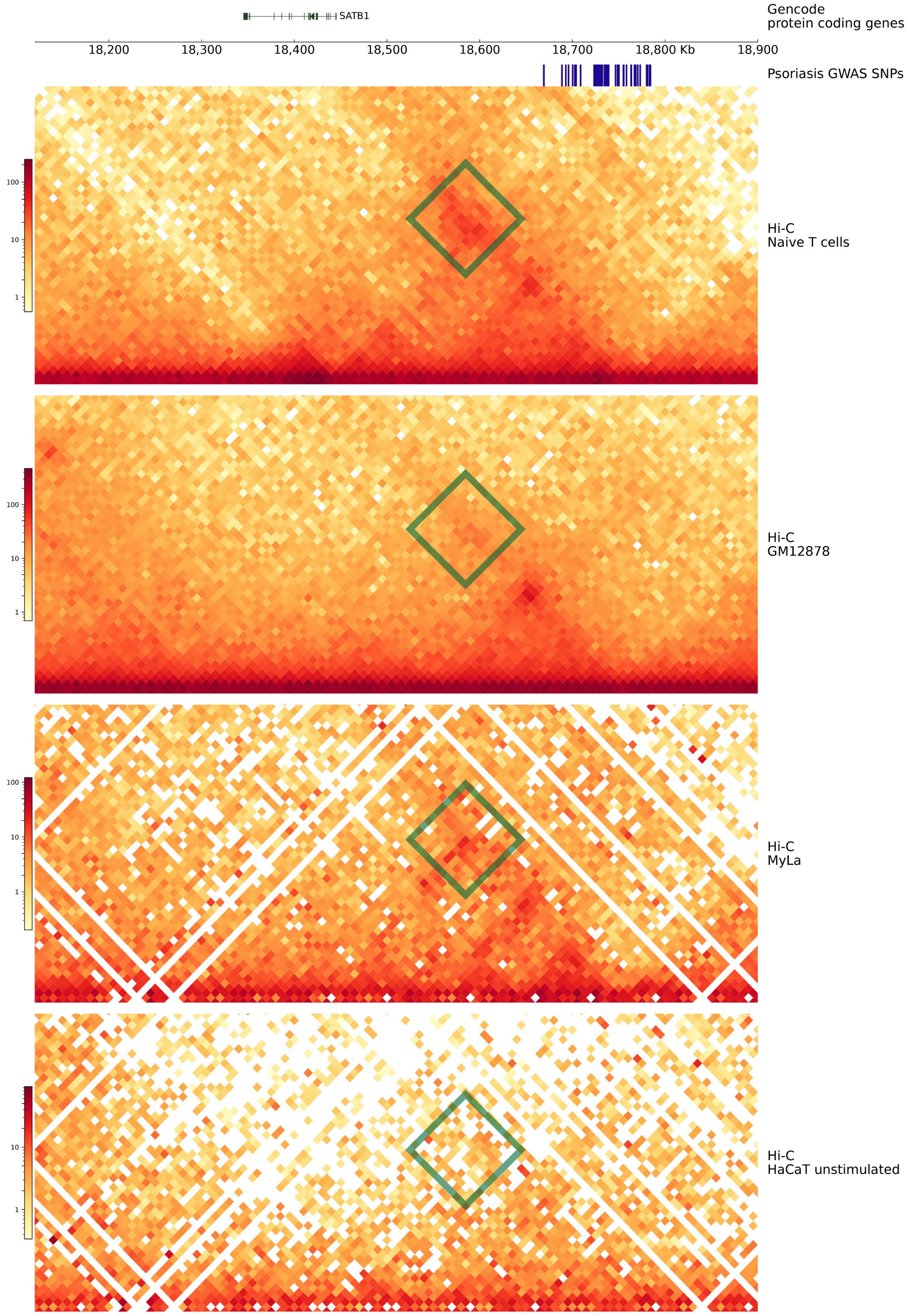

Figure S12. HiChIP interactions from the *EXOC2/IRF4/DUSP22* locus link *IRF4* and *DUSP22* as candidate genes in psoriasis. Tracks (in order): Gencode genes; SNPs associated with Psoriasis (r^2 > 0.8); H3K27ac signal in naïve T cells; H3K27ac signal in MyLa cells; Significant long range interactions originating from the atopic dermatitis associated locus in MyLa cells; H3K27ac signal in unstimulated HaCaT cells; H3K27ac signal in IFN-γ stimulated HaCaT cells; H3K27ac signal in GM12878 cells; H3K27ac signal in naïve T cells.

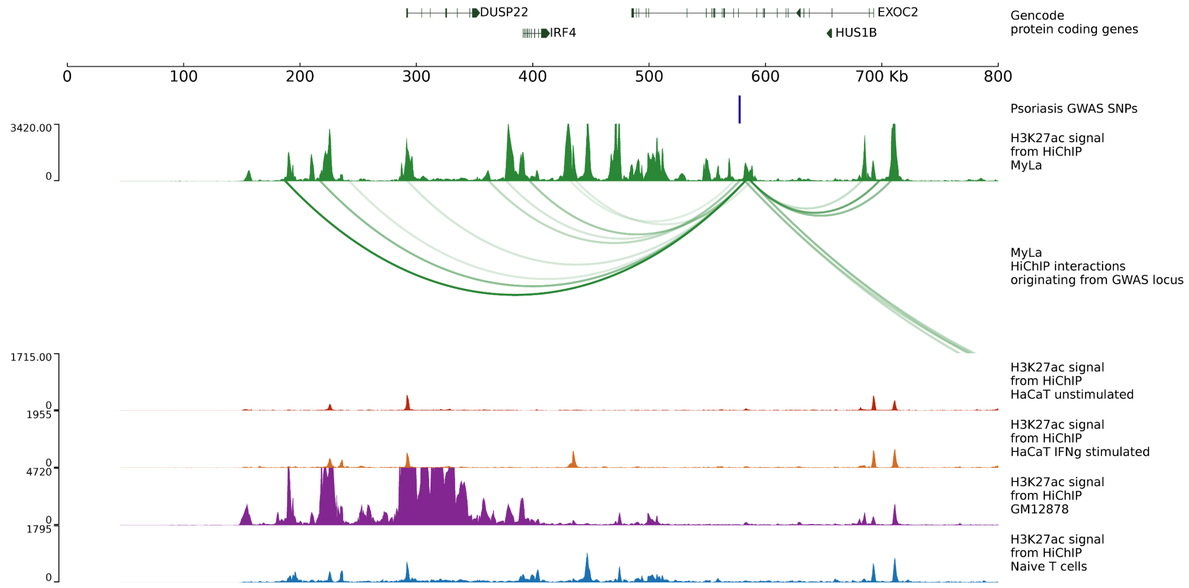

Figure S13. Hi-C contact maps for the EXOC2/IRF4/DUSP22 locus.

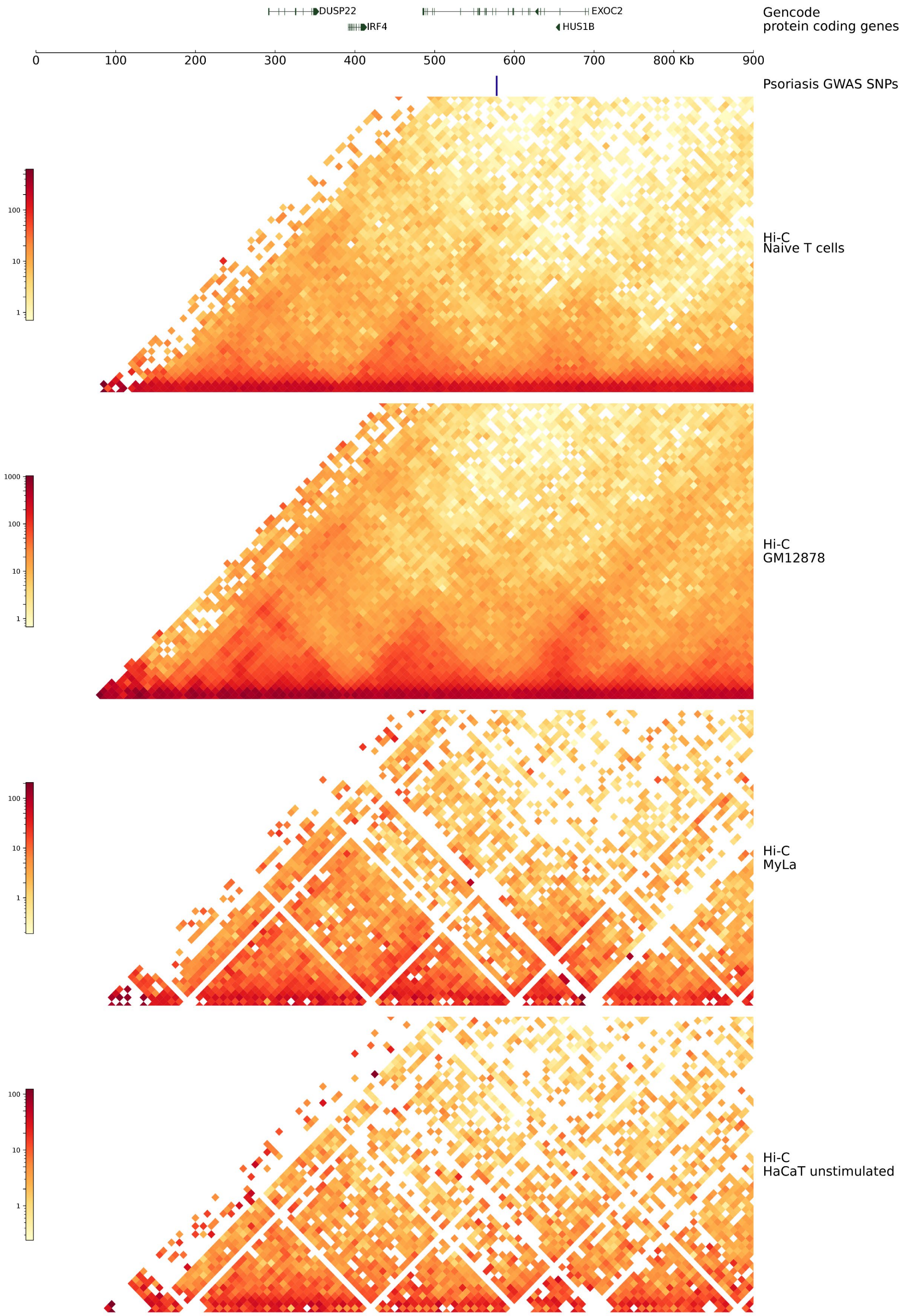

Figure S14. HiChIP interactions from the IFNLR1/GRHL3 locus link GRHL3 as a candidate gene in psoriasis. Tracks for other cell types. Tracks (in order): RefSeq genes; SNPs associated with Psoriasis; H3K27ac signal in GM12878 cells; Significant long range interactions originating from the psoriasis associated locus in GM12878 cells; H3K27ac signal in MyLa cells; Significant long range interactions originating from the psoriasis associated locus in MyLa cells; H3K27ac signal in Naïve T cells; Significant long range interactions originating from the psoriasis associated locus in Naïve T cells.

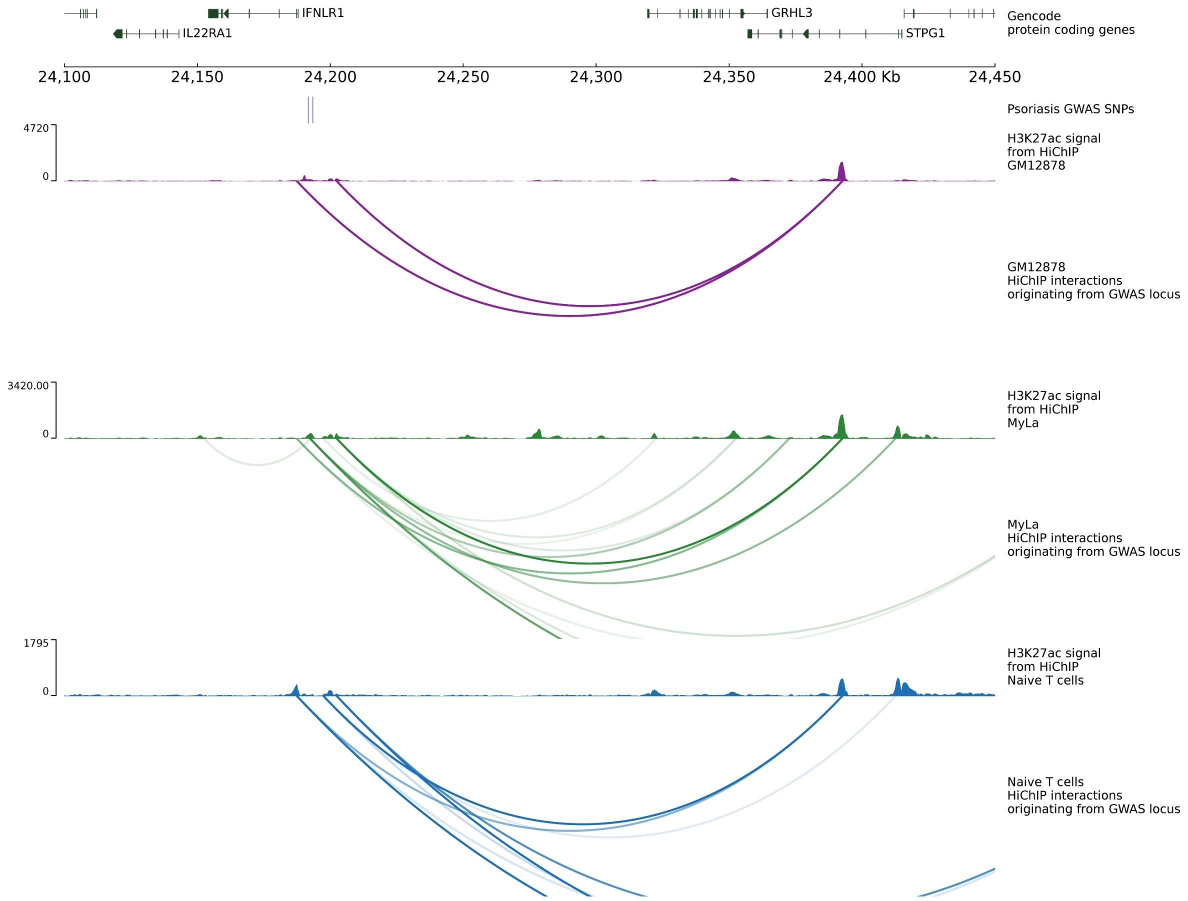

Figure S15. Hi-C contact maps for the IFNLR1/GRHL3 locus.

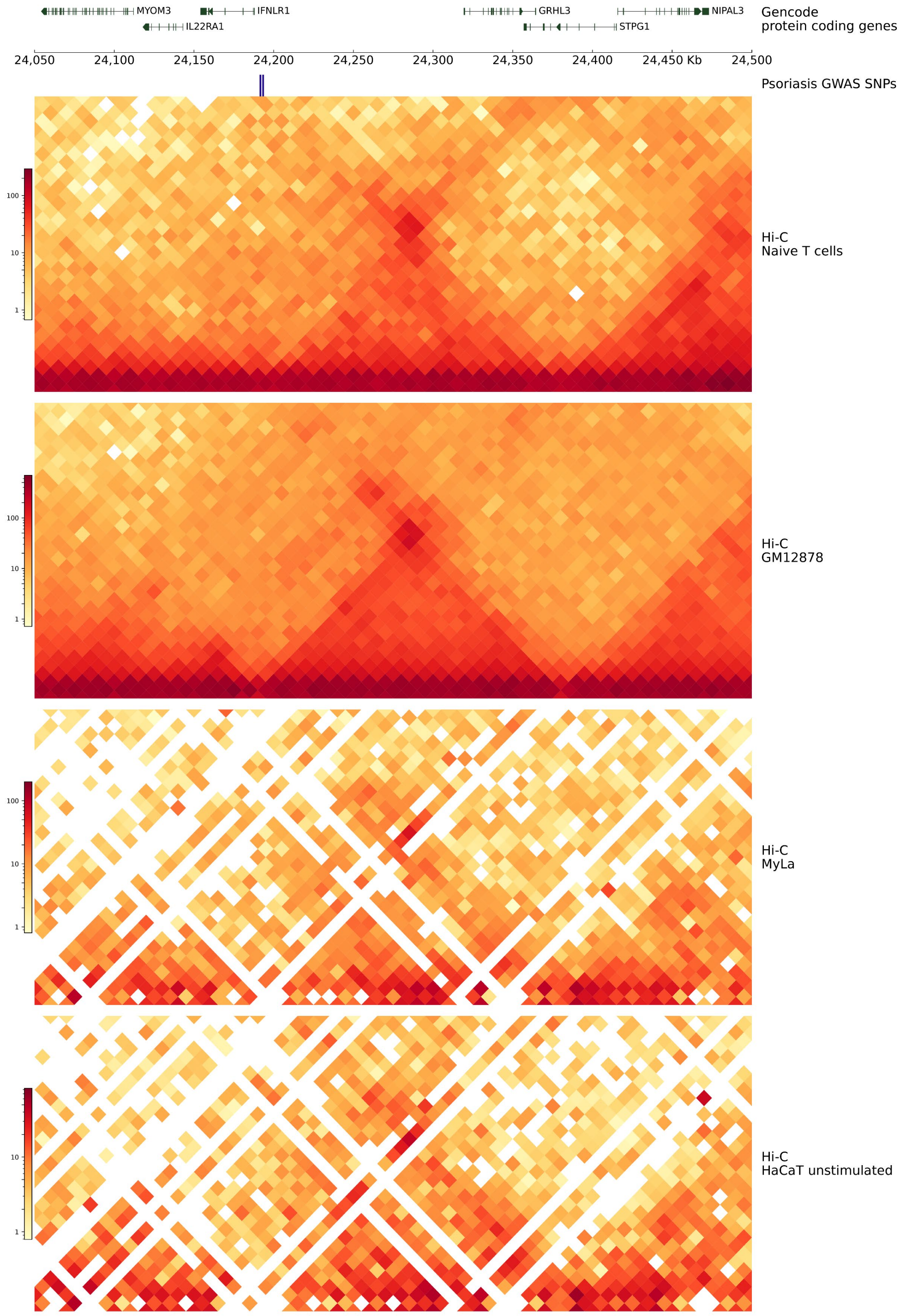

Figure S16. Comparison of H3K27ac in HaCaT cells with publicly available ChIP-seqs generated from primary keratinocytes in different conditions. A) View of the promoter region of GRHL3. all conditions show a peak for H3K27ac and H3K4me3 indicating promoter activity for this gene in all conditions. B) View of the GWAS SNPs. We can see that there is presence of H3K27ac and H3K4me1 in progenitor cells and H3K4me1 in migrating cells but no H3K4me3 in any condition. This indicates that the region functions as an enhancer and not as a promoter (which is next to it rather than overlapping) in these cell populations.

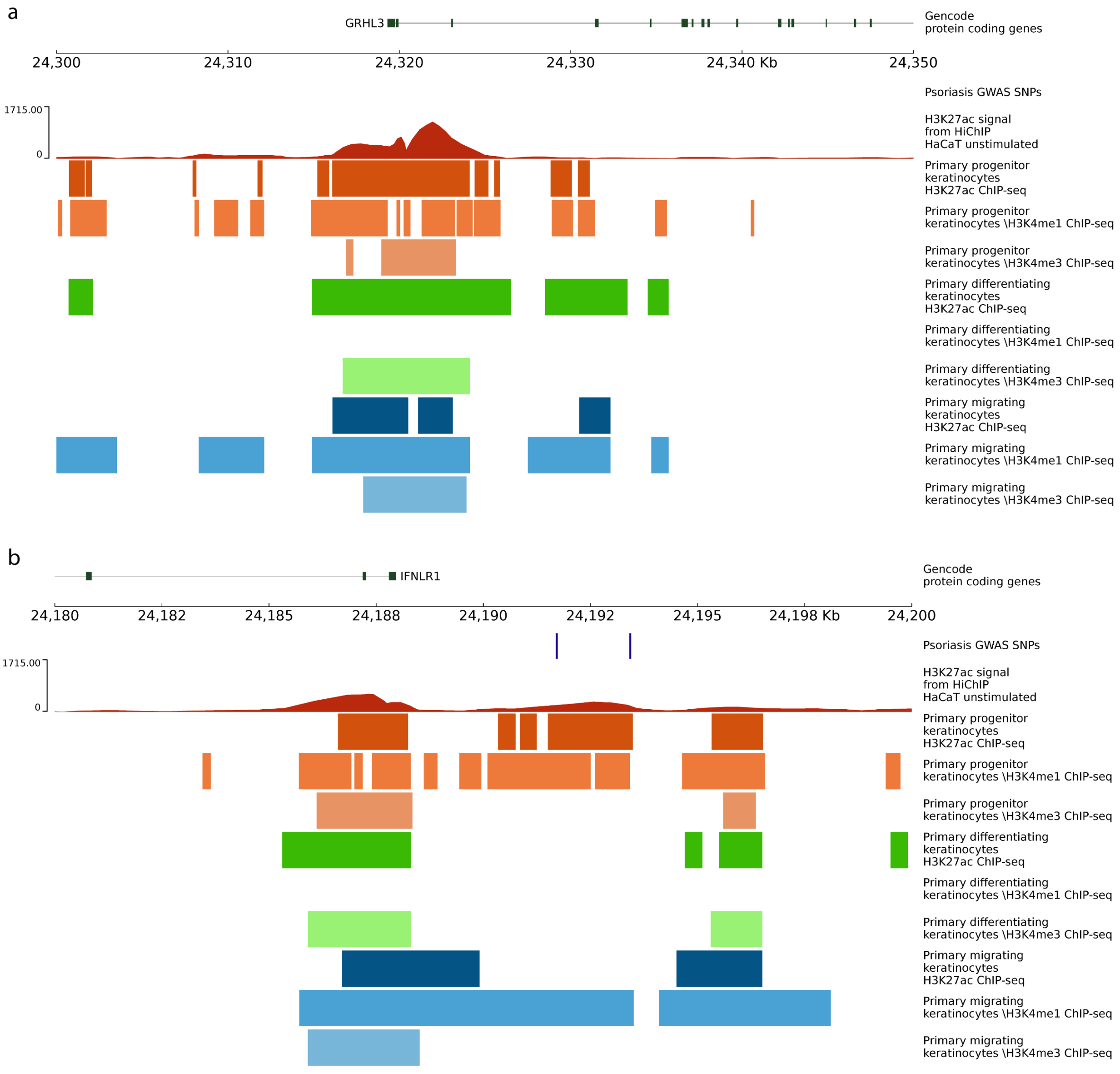

Figure S17. Hi-C contact maps for the FOXO1 locus.

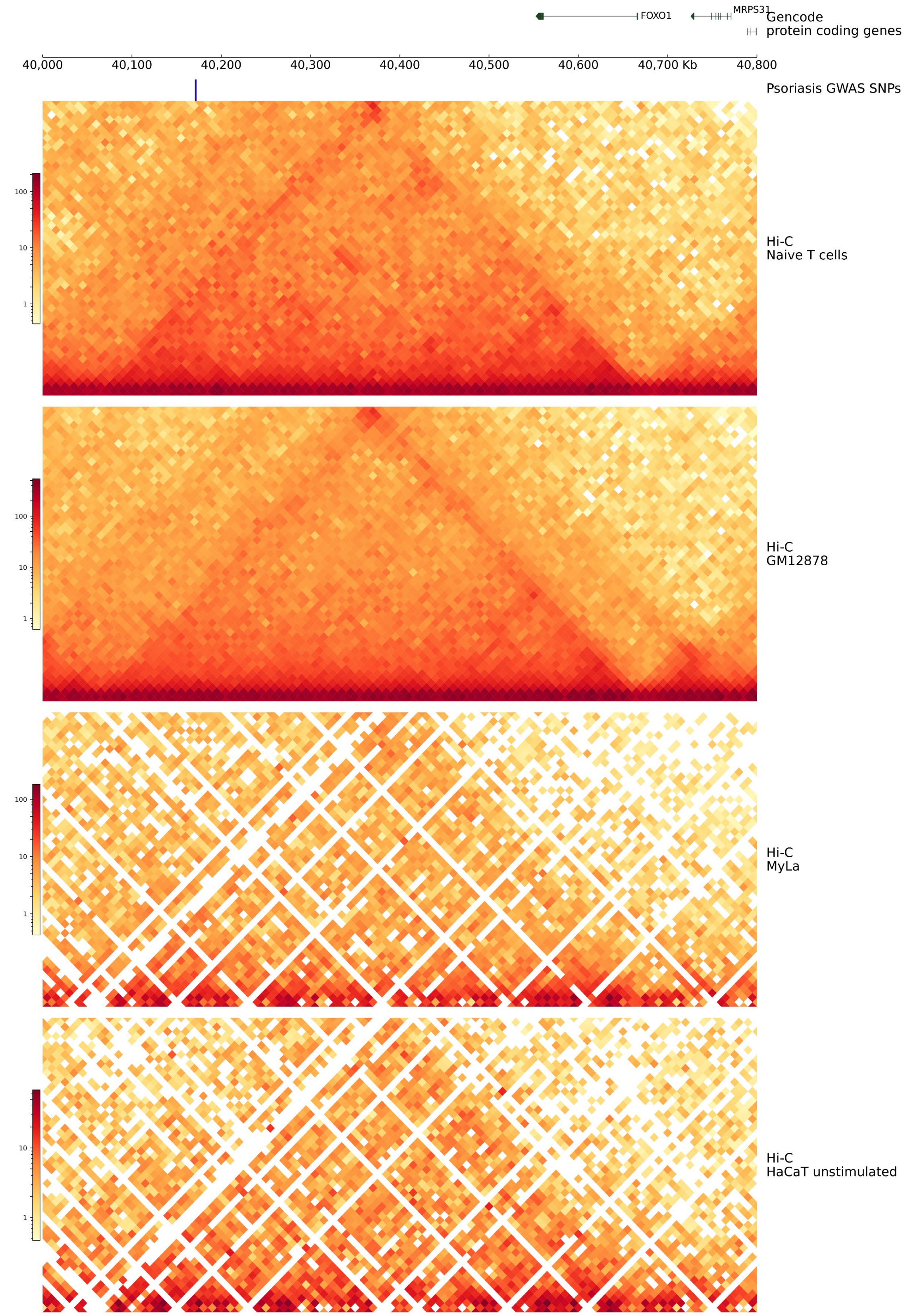

Supplementary materials and methods

Cell culture

HaCaT keratinocyte cells (Addexbio T0020001) and My-La CD8+ cells (Sigma-Aldrich, 95051033) were authenticated by STR DNA profiling at the University of Manchester. HaCaT cells were cultured in high-glucose Dulbecco's modified eagle's medium (DMEM) supplemented with 10% foetal bovine serum (FBS) and penicillin-streptomycin. For HaCaT stimulation experiments, the media was supplemented with 100 ng/mL recombinant human IFN-γ (285-IF-100; R&D Systems) and cells incubated for 8 hours prior to harvest. My-La CD8+ cells (Sigma-Aldrich, 95051033) were cultured in Roswell Park Memorial Institute (RPMI) 1640 medium supplemented with 10% AB human serum (Sigma Aldrich), 100 U/mL recombinant human IL-2 (Sigma-Aldrich) and penicillin-streptomycin (final concentration 100 U penicillin, 0.1 mg streptomycin/ml). HaCaT and My-La cells were tested for mycoplasma by enzymatic assay at the University of Manchester (last tested on 29/10/2020).

Cell crosslinking for chromatin-based experiments

HaCaT cells were crosslinked for 10 minutes in 1% formaldehyde and the reaction was quenched with 0.135M glycine. The crosslinked cells were scraped from the flask, pelleted, washed in PBS and the supernatant removed. My-La cells were crosslinked for 10 minutes in 1% (HiChIP) or 2% (Hi-C) formaldehyde and the reaction was quenched with 0.135M glycine and the supernatant removed. Fixed cells were snap frozen on dry ice and stored at -80°C.

HiChIP library generation and processing

HiChIP libraries were generated according to the Chang Lab protocol (Mumbach et al., 2016). 10 million crosslinked cells were lysed and the chromatin digested using 375 U of MboI (NEB, R0147M) for 4 hours at 37°C. Fragment ends were filled in using dCTP, dGTP, dTTP and biotin-14 dATP (Life Technologies) and ligated at room temperature overnight. The nuclei were lysed and the chromatin sheared to lengths of approximately 200-700 bp using a Covaris S220. Immunoprecipitation was performed overnight at 4°C using 20 µg of H3K27ac antibody (Abcam ab4729). The DNA was captured on a 1:1 mixture of protein A and G Dynabeads (Invitrogen 10001D and 10003D). After washes, the DNA was eluted with proteinase K at 65°C overnight. The sample was cleaned using Zymo Clean and Concentrator Columns (Zymo D4013) and quantified using the Qubit DNA HS kit. 20-35 ng of DNA was taken forward for biotin-pulldown with streptavidin C-1 beads at room temperature for 30 minutes. The beads were suspended in TD buffer from the Nextera kit and transposed with Tn5 (Illumina) at 55°C for exactly 10 minutes. The volume of Tn5 was dependent on DNA quantity and defined by the original HiChIP protocol. After washes, the library was amplified off the beads using Phusion polymerase and Nextera indexing primers (Illumina). AMPure XP beads (Beckman Coulter, A63882) were used to select fragments approximately 300 – 700 bp in length. Quantification and quality control of the final HiChIP library was conducted using a Bioanalyzer and KAPA quantification kit (Kapa Biosystems). Libraries underwent Next Generation Sequencing on a HiSeq 2500 generating 100 bp paired-ends.

Sequencing data for the HiChIP libraries was filtered and the adapters were removed using fastp v0.19.4 (Chen et al., 2018). The reads were then mapped to the GRCh38 genome with Hi-C Pro v2.11.0 (Servant et al., 2015), using default settings. Enriched regions (H3K27ac peaks) were identified using HiChIP-peaks v 0.1.1 (Shi et al., 2019) with default settings and FDR < 0.01. Loops were identified using FitHiChIP (Bhattacharyya et al., 2019) using the following settings: Coverage normalization, stringent background with merging enabled,

peaks generated from HiChIP-peaks and 5 kb bin size. Viewpoints for the figures were generated by selecting interactions originating from within 10 kb of the SNPs.

We have noticed that one replicate produced significantly more loops than the other replicate. This could be caused by the fact that this replicate contained significantly more reads than the latter. We tested this hypothesis by down sampling the number of unique valid pairs identified in one replicate (HaCaT unstim 2) to the same depth of the other (HaCaT unstim 1). While this reduced the number of loops identified slightly it was not enough by itself to correct the number of loops (from 38118 to 36211). The results from HiChIP-peaks show that one replicate 2 had significantly higher proportion of reads in peaks (a metric for ChIP enrichment) compared to replicate 1 (54% vs 45%). In addition to this we also noticed some slight differences in the raw contact statistics in which replicate 2 contained more short range (<20kb) paired end tags than replicate 1 (30% vs 15%) and fewer long range and trans paired end tags (trans: 13% vs 20%, >20kb: 56% vs 63%). We think that these two factors could be the main contributors to the differences in number of loops called and the PCA in the next section, rather than the number of reads.

To create the Venn diagrams for the overlap of the interactions we used bedtools v2.28.0 (Quinlan & Hall, 2010) PairToPair with a slop of 5kb to identify the interactions that were present in two conditions, which was then repeated for each two-way comparison between the replicates and the combined datasets. Because of the slop some interactions in one group could overlap multiple interactions from the other group and vice-versa which can cause some inconsistencies in the reported numbers in which the sum of all subsections of a group is higher than the total number of interactions. We have tried to minimize this effect, but the results remain approximate. This does not affect the overall message of the figures presented.

HiChIP clustering and principal component analysis

To validate the reproducibility and cell type specificity of our HiChIP loops we collected the top 10000 significant loops from each individual replicate and combined it to create a set of 82545 loops across all samples. For each of these loops we then collected the raw FitHiChIP p-value from each sample from the raw interactions file (replacing missing entries with 1). We then ran hierarchical clustering using correlation (seaborn clustermap) and PCA (Scikit-learn) analysis on the resulting data matrix.

For the clustering of the peaks we used the included differential peak calling module of HiChIP-peaks (Shi et al., 2019) and used the data matrix provided to run hierarchical clustering using correlation (seaborn clustermap) and PCA (Scikit-learn) analysis on the resulting data matrix.

We noticed that although the hierarchical clustering showed a better correlation between conditions than across batches, the PCA showed that the amount variance explained by batch was greater than the IFN-γ stimulation. We see this both when analysing loops or peaks by themselves (figure 1A-B, S1A-B). We believe that slight differences in culturing conditions introduced this variability as these are biological replicates that have been generated completely independently of each other. The results from differential analysis of both RNA-seq and peaks' motifs enrichments show strong enrichment for IFN-γ response pathways, indicating that the stimulation was nevertheless successful and created the right response in the samples.

Hi-C library generation and processing

In-situ Hi-C libraries were generated for HaCaT and MyLa cell lines as previously described (Ray-Jones et al., 2020). 50 million crosslinked cells were lysed and the chromatin digested with HindIII at 37°C overnight. Restriction cut sites were filled in using dCTP, dGTP, dTTP and biotin-14-dATP (Life Technologies), then in-nucleus ligation was carried out at 16°C for 4-6 hours. Crosslinks were reversed by proteinase-K overnight at 65°C and RNA was digested using RNaseA for 60 minutes at 37°C. The DNA was purified by sequential phenol and phenol-chloroform extractions and ethanol-precipitated at -20°C overnight, followed by two further phenol-chloroform extractions and a second overnight precipitation.

A 40 µg aliquot of DNA was taken forward for further processing following QC steps. T4 DNA polymerase used to remove biotin-14-dATP from non-ligated ends then the DNA purified by phenol-chloroform extraction and ethanol precipitation overnight. The DNA was sheared using a Covaris S220 sonicator and end-repair was performed using T4 DNA polymerase, T4 DNA polynucleotide kinase and DNA polymerase I, large (Klenow) fragment. The sample was purified using Qiagen MinElute Kit, with a modified protocol described by (Belton et al., 2012). Klenow (exo-) was used to adenylate DNA fragment ends and a double-sided SPRI bead size selection was used to obtain fragments of approximately 200-600 bp. Dynabeads MyOne Streptavidin C1 beads (Life Technologies) were used to pull down biotinylated fragments, which were then ligated to annealed Illumina sequencing adapters. PCR was performed using Phusion HF (NEB) and TruPE PCR primers (Illumina), then the amplified DNA was cleaned twice using 1.8X volume of SPRI beads. The quality and quantity of the Hi-C libraries was tested by Bioanalyzer and KAPA qPCR. Hi-C libraries were analysed by Next Generation Sequencing. The My-La Hi-C library was sequenced on an Illumina HiSeq 2500 generating 100bp paired ends. The HaCaT Hi-C libraries were sequenced on an Illumina HiSeq 4000 generating 75 bp paired ends.

The sequencing data was filtered and adapters were removed using fastp v0.19.4 (Chen et al., 2018). The reads were then mapped to the GRCh38 genome with Hi-C Pro v2.11.0 (Servant et al., 2015), using default settings. The Hi-C interaction matrices were normalised within Hi-C Pro using iterative correction and eigenvector decomposition (ICE). TADs were identified using OnTAD v1.2 (An et al., 2019), a novel Optimized Nested TAD caller for Hi-C data, using Hi-C data binned at a 40 kb resolution and a maximum TAD size of 4 mb. This software calls TADs using a hierarchical algorithm. Level 1 TADs are the biggest and within each TAD there can be smaller subTADs which are identified as level 2, 3 etc. For this analysis we only used the first 3 levels. Files for visualisation were created using the hicpro2juicebox.sh utility and visualised in Juicebox (Durand et al., 2016). Maps were normalized with the balancing algorithm whenever that converged or the coverage (sqrt) method otherwise. Figures for this paper were generated using a modified version of CoolBox (https://github.com/GangCaoLab/CoolBox).

For primary Naïve T cells, we used a different protocol to generate the Hi-C maps. PBMCs were isolated from a buffy coat obtained from the National Blood Transfusion Service using a ficoll gradient. T-cells were isolated using an EasySep T-cell isolation kit according to the manufacturer's instructions. 3 million cells were fixed with 2% formaldehyde for 10 minutes and then snap frozen. Hi-C libraries for 3 technical replicates were generated using the Arima Hi-C kit following manufacturer's instructions. Data was then merged, processed and analysed in the same way as the other libraries.

For GM12878 cells we obtained 1.2B raw reads from Arima Genomics. This library was chosen because it was more directly comparable with our primary Naïve T cells library and, as it was generated using the Arima Hi-C protocol. Data was then processed and analysed in the same way as the other libraries.

Region Capture Hi-C and overlap with HiChIP results

Region capture Hi-C libraries were generated as previously described from the Hi-C libraries as part of a previous study (Ray-Jones et al., 2020). Approximately 4500 baits were used that captured fragments containing GWAS SNPs for Ps, juvenile idiopathic arthritis, asthma, PsA, rheumatoid arthritis and SSc.

First, amplified Hi-C libraries were generated as above. Then, Hi-C DNA up to 750 ng was concentrated using a vacuum concentrator and bound to the capture baits in a single hybridisation reaction using SureSelectXT reagents and protocol (Agilent Technologies). The biotinylated baits were captured using Dynabeads MyOne Streptavidin T1 beads (Life Technologies). Following washes, the libraries were amplified on the beads using Phusion HF and barcoded TruPE primers then the amplified DNA cleaned twice using 1.8X volume of SPRI beads. The quality and quantity of the capture Hi-C libraries was tested by Bioanalyzer and KAPA qPCR (Kapa Biosystems). Capture Hi-C libraries were analysed by 75 bp paired-end Next Generation Sequencing on an Illumina NextSeq500 (My-La) or HiSeq 4000 (HaCaT).

Capture Hi-C sequence data was quality filtered with fastp v 0.19.4 (Chen et al., 2018) and then processed through the Hi-C User Pipeline (HiCUP) v0.7.2 (Wingett et al., 2015) and mapped to the GRCh38 genome. For each cell type, the two biological replicates were simultaneously run through Capture Hi-C Analysis of Genomic Organisation (CHiCAGO) v1.10.1 (Cairns et al., 2016) in R v3.5.1 and significant interactions were called with a score threshold of 5. This yielded about 35,000 significant interactions across all disease studied, with a median interaction distance of 280 kb (figure 1C and S4B). Similar to HiChIP data, the majority of the interactions reside within TADs (figure 1D).

Enrichment for H3K27ac was calculated using the integrated tool in the CHiCAGO package with default settings and the H3K27ac peaks generated from the HiChIP data using HiChIP-Peaks.

To identify active enhancer-promoter interactions we kept the CHiCAGO interactions that originated from HiChIP H3K27ac peaks in the matching cell type. We then identified the expressed promoters (TPM>1) that were within 5 kb of the other end of the interactions.

To compare the results from Capture Hi-C with our new HiChIP libraries we determined the interactions that originated from within 5 kb of those 4500 capture fragments. Genes were then identified as described in the "Linking GWAS results to putative gene targets" section.

Even though Capture Hi-C interactions are enriched for H3K27ac (figure S5B), they are not specifically selected for active genes or enhancers. The majority of significant interactions (80% for unstimulated HaCaT) do not overlap H3K27ac peaks. Moreover, Capture Hi-C interactions do not seem specific for active genes: 36% of the genes interacting with baits in unstimulated HaCaT are not expressed. In contrast, HiChIP interactions overlap a H3K27ac peak 99.8% of the times at one end and 92.5% at both ends. Moreover, 82% of the interacting genes from the same regions were found to be expressed.

RNA-seq

3' mRNA sequencing libraries were generated for cell lines using the Lexogen QuantSeq 3' mRNA-Seq Library Prep Kit FWD for Illumina. Libraries were sequenced using single-end Illumina SBS technology. Reads were quality trimmed using Trimmomatic v0.38 (Bolger et al., 2014) using a sliding window of 5 with a mean minimum quality of 20. Adapters and poly A/poly G tails were removed using Cutadapt v1.18 (Martin, 2011) and then UMIs were extracted from the 5' of the reads using UMI-tools v0.5.5 (Smith et al., 2017). Reads were then mapped using STAR v2.5.3a (Dobin et al., 2013) on the GRCh38 genome with GENCODE annotation v29 (Harrow et al., 2012). Reads were de-duplicated using UMIs with UMI-tools and then counted using HTSeq v0.11.2 (Anders et al., 2015). Count matrixes were analysed in R 3.5.1 and normalisation and differential expression analysis was conducted using DESeq2 v1.22.2 (Love et al., 2014). Differentially expressed genes were called with an adjusted P value of 0.10 (FDR 10%). For detection of expressed genes in the cell lines, we considered RNA-seq counts greater than 1 count per million.

Public RNA-seq data

Public RNA-seq for the CD4 naïve t cell type was downloaded from (Bonnal et al., 2015). Accession ID: ERP004883. Raw sequencing reads were filtered and adapters and polyAs trimmed with fastp v 0.19.4 (Chen et al., 2018). Reads were then mapped with salmon v0.14.1 (Patro et al., 2017) to the GRCh38 genome with GENCODE annotation v29 (Harrow et al., 2012).

TPM values were used later in the analysis for gene filtering.

GWAS data

Genome wide significant (p-value < 5x10^-8) GWAS loci were downloaded for the following diseases: PsA (Bowes et al., 2015; Stuart et al., 2015), Ps (Tsoi et al., 2017), melanoma (Duffy et al., 2018), SSc (López-Isac et al., 2019), atopic dermatitis (Paternoster et al., 2015) and rheumatoid arthritis (Okada et al., 2014). GWAS loci for Skin Pigmentation (Hernandez-Pacheco et al., 2017), Celiac Disease (Trynka et al., 2011), Grave's Disease (Cooper et al., 2012), Primary Biliary Cholangitis (Cordell et al., 2015), Type 2 Diabetes (Mahajan et al., 2018), Chrohn Disease (Liu et al., 2015), Keratinocyte carcinoma (Liyanage et al., 2019) and Primary Sclerosing Cholangitis (Ji et al., 2017) (used in supplementary figures only) were downloaded from the GWAS catalog (Buniello et al., 2019).

SNPs in high linkage disequilibrium (R^2 > 0.8) with the lead SNPs were identified using plink v1.90b3.39 on the 1000 genomes data v3 with population set to EUR.

SNP enrichment

We obtained the H3K27ac signal tracks for each cell type from the HiChIP data using HiChIP-Peaks. This track corresponds to the signal for this marker of activity along the genome. We then calculated the median intensity of the signal over every SNP outside of the MHC and compared it with the median for a set of 1 million randomly generated positions to get an estimate of a genomic background for the signal to calculate an enrichment. We also calculated the number of individual SNPs that are located within a H3K27ac peak for each cell type.

Linking stimulation responsive genes to enhancers and motifs

We first identified the genes that were differentially expressed during the IFN-γ stimulation as described previously. We then used the promoter regions of these genes to identify the

regions that interacted with these genes in the two conditions in HaCaT cells assuming these regions would be enhancers that regulated these genes.

To test the levels of H3K27 acetylation present in these peaks before and after the IFN-γ stimulation we intersected these regions with the H3K27ac peaks identified from HiChIP-Peaks and recorded their normalized H3K27ac signal.

To identify the enriched motifs that were linked to differentially expressed genes we obtained the H3K27ac peak regions that were linked in both conditions to these genes and ran Motif enrichment analysis using HOMER v 4.8.3 (Heinz et al., 2010) with the findMotifsGenome.pl command and "–size given" parameter. The background model was set to all the peaks identified in the two conditions.

To identify the enriched motifs in peaks linked to UP regulated genes specifically in IFN-γ stimulated condition we first identified the regions (other ends) that were linked to these genes in the two conditions. We then used bedtools v2.28.0 (Quinlan & Hall, 2010) to identify the other ends that were linked in IFN-γ stimulated condition but not in unstimulated condition with a slop of 5kb. We then intersected those regions with the H3K27ac peaks as done previously and ran findMotifsGenome.pl command with the "–size given" parameter. The background model was set to all the peaks that were linked to these same genes in both conditions.

The motifs database used was always the default HOMER Known motifs database and all other settings for HOMER were kept to default.

Overlap with eQTL

We downloaded the full cis-eQTL datasets from the sun-exposed skin GTEx v7 dataset (GTEx Consortium, 2013), the eQTLgen (version 2018/10/17) dataset (Võsa et al., 2018), the Kasela dataset (Kasela et al., 2017) and the DICE eQTL dataset (Schmiedel et al., 2018). To identify the eQTLs that were originating from the GWAS loci we queried every SNP that was in LD ($R^2 > 0.8$) with the lead SNP and recorded all the genes that were significantly linked to those variants. Genes were filtered by expression TPM > 1 in our cell types. We then identified all the genes that are linked for a specific disease (in this example psoriasis) and compared this list with the list of genes that were identified from the same SNPs using the HiChIP interactions.

Pathway analysis

The most enriched pathways for each disease were identified using the EnrichR (E. Y. Chen et al., 2013) web API with the gene set library set to GO_Biological_Process_2018 with all protein coding genes as background (default settings). We used the genes that were called in both replicates for each condition for this analysis. The pathways were then sorted by p-value and the top 10 enriched pathways were plotted.

Loci style

To classify loci into general groups of interactions we first identified the closest genes to the loci. A locus can have multiple closest genes as the LD block might span a large region and multiple genes could be considered the "closest" to some SNP.

To identify the number of loci in which we link all or some of the closest genes we first decided to remove the loci which had an LD block larger than 100kb. We then tested if our interactions (from merged datasets) recovered these genes. To identify the loci which did not have any

close gene we did not filter by loci size although none of the loci tested was above 100kb in size.

Drug discovery

Drug discovery was executed by querying the drugbank v5.1.4 database (Wishart et al., 2008). New drugs available for repurposing were identified by the approved tag in at least one disease and the target being one of the genes studied.

REFERENCES

An, L., Yang, T., Yang, J., Nuebler, J., Xiang, G., Hardison, R. C., Li, Q., & Zhang, Y. (2019). Hierarchical Domain Structure Reveals the Divergence of Activity among TADs and Boundaries. *BioRxiv*, 361147. https://doi.org/10.1101/361147

Anders, S., Pyl, P. T., & Huber, W. (2015). HTSeq-A Python framework to work with high-throughput sequencing data. *Bioinformatics*, *31*(2), 166–169. https://doi.org/10.1093/bioinformatics/btu638

Belton, J. M., McCord, R. P., Gibcus, J. H., Naumova, N., Zhan, Y., & Dekker, J. (2012). Hi-C: A comprehensive technique to capture the conformation of genomes. *Methods*, *58*(3), 268–276. https://doi.org/10.1016/j.ymeth.2012.05.001

Bhattacharyya, S., Chandra, V., Vijayanand, P., & Ay, F. (2019). Identification of significant chromatin contacts from HiChIP data by FitHiChIP. *Nature Communications*, *10*(1). https://doi.org/10.1038/s41467-019-11950-y

Bolger, A. M., Lohse, M., & Usadel, B. (2014). Trimmomatic: A flexible trimmer for Illumina sequence data. *Bioinformatics*, *30*(15), 2114–2120. https://doi.org/10.1093/bioinformatics/btu170

Bonnal, R. J. P., Ranzani, V., Arrigoni, A., Curti, S., Panzeri, I., Gruarin, P., Abrignani, S., Rossetti, G., & Pagani, M. (2015). De novo transcriptome profiling of highly purified human lymphocytes primary cells. *Scientific Data*, *2*(1), 150051. https://doi.org/10.1038/sdata.2015.51

Bowes, J., Budu-Aggrey, A., Huffmeier, U., Uebe, S., Steel, K., Hebert, H. L., Wallace, C., Massey, J., Bruce, I. N., Bluett, J., Feletar, M., Morgan, A. W., Marzo-Ortega, H., Donohoe, G., Morris, D. W., Helliwell, P., Ryan, A. W., Kane, D., Warren, R. B., … Barton, A. (2015). Dense genotyping of immune-related susceptibility loci reveals new insights into the genetics of psoriatic arthritis. *Nature Communications*, *6*, 6046. https://doi.org/10.1038/ncomms7046

Buniello, A., Macarthur, J. A. L., Cerezo, M., Harris, L. W., Hayhurst, J., Malangone, C., McMahon, A., Morales, J., Mountjoy, E., Sollis, E., Suveges, D., Vrousgou, O., Whetzel, P. L., Amode, R., Guillen, J. A., Riat, H. S., Trevanion, S. J., Hall, P., Junkins, H., … Parkinson, H. (2019). The NHGRI-EBI GWAS Catalog of published genome-wide association studies, targeted arrays and summary statistics 2019. *Nucleic Acids Research*, *47*(D1), D1005–D1012. https://doi.org/10.1093/nar/gky1120

Cairns, J., Freire-Pritchett, P., Wingett, S. W., Várnai, C., Dimond, A., Plagnol, V., Zerbino, D., Schoenfelder, S., Javierre, B.-M., Osborne, C., Fraser, P., & Spivakov, M. (2016). CHiCAGO: robust detection of DNA looping interactions in Capture Hi-C data. *Genome Biology*, *17*(1), 127. https://doi.org/10.1186/s13059-016-0992-2

Chen, S., Zhou, Y., Chen, Y., & Gu, J. (2018). fastp: an ultra-fast all-in-one FASTQ preprocessor. *Bioinformatics*, *34*(17), i884–i890. https://doi.org/10.1093/bioinformatics/bty560

Cooper, J. D., Simmonds, M. J., Walker, N. M., Burren, O. S., Brand, O. J., Guo, H., Wallace, C., Stevens, H., Coleman, G., Franklyn, J. A., Todd, J. A., Gough, S. C. L., Aerts, J., Ahmad, T., Arbury, H., Attwood, A., Auton, A., Ball, S. G., Balmforth, A. J., … Donnelly, P. (2012). Seven newly identified loci for autoimmune thyroid disease. *Human Molecular Genetics*, *21*(23), 5202–5208. https://doi.org/10.1093/hmg/dds357

Cordell, H. J., Han, Y., Mells, G. F., Li, Y., Hirschfield, G. M., Greene, C. S., Xie, G., Juran, B. D., Zhu, D., Qian, D. C., Floyd, J. A. B., Morley, K. I., Prati, D., Lleo, A., Cusi, D., Gershwin, M. E., Anderson, C. A., Lazaridis, K.

N., Invernizzi, P., … Kordula, J. (2015). International genome-wide meta-analysis identifies new primary biliary cirrhosis risk loci and targetable pathogenic pathways. *Nature Communications*, *6*(1), 1–11. https://doi.org/10.1038/ncomms9019

Dobin, A., Davis, C. A., Schlesinger, F., Drenkow, J., Zaleski, C., Jha, S., Batut, P., Chaisson, M., & Gingeras, T. R. (2013). STAR: Ultrafast universal RNA-seq aligner. *Bioinformatics*, *29*(1), 15–21. https://doi.org/10.1093/bioinformatics/bts635

Duffy, D. L., Zhu, G., Li, X., Sanna, M., Iles, M. M., Jacobs, L. C., Evans, D. M., Yazar, S., Beesley, J., Law, M. H., Kraft, P., Visconti, A., Taylor, J. C., Lui, F., Wright, M. J., Henders, A. K., Bowdler, L., Glass, D., Ikram, A. M., … Martin, N. G. (2018). Novel pleiotropic risk loci for melanoma and nevus density implicate multiple biological pathways. *Nature Communications*, *9*(1). https://doi.org/10.1038/s41467-018-06649-5

Durand, N. C., Robinson, J. T., Shamim, M. S., Machol, I., Mesirov, J. P., Lander, E. S., & Aiden, E. L. (2016). Juicebox Provides a Visualization System for Hi-C Contact Maps with Unlimited Zoom. *Cell Systems*, *3*(1), 99–101. https://doi.org/10.1016/j.cels.2015.07.012

GTEx Consortium. (2013). The Genotype-Tissue Expression (GTEx) project. *Nature Genetics*, *45*(6), 580–585. https://doi.org/10.1038/ng.2653

Harrow, J., Frankish, A., Gonzalez, J. M., Tapanari, E., Diekhans, M., Kokocinski, F., Aken, B. L., Barrell, D., Zadissa, A., Searle, S., Barnes, I., Bignell, A., Boychenko, V., Hunt, T., Kay, M., Mukherjee, G., Rajan, J., Despacio-Reyes, G., Saunders, G., … Hubbard, T. J. (2012). GENCODE: The reference human genome annotation for the ENCODE project. *Genome Research*, *22*(9), 1760–1774. https://doi.org/10.1101/gr.135350.111

Heinz, S., Benner, C., Spann, N., Bertolino, E., Lin, Y. C., Laslo, P., Cheng, J. X., Murre, C., Singh, H., & Glass, C. K. (2010). Simple Combinations of Lineage-Determining Transcription Factors Prime cis-Regulatory Elements Required for Macrophage and B Cell Identities. *Molecular Cell*, *38*(4), 576–589. https://doi.org/10.1016/J.MOLCEL.2010.05.004

Hernandez-Pacheco, N., Flores, C., Alonso, S., Eng, C., Mak, A. C. Y., Hunstman, S., Hu, D., White, M. J., Oh, S. S., Meade, K., Farber, H. J., Avila, P. C., Serebrisky, D., Thyne, S. M., Brigino-Buenaventura, E., Rodriguez-Cintron, W., Sen, S., Kumar, R., Lenoir, M., … Pino-Yanes, M. (2017). Identification of a novel locus associated with skin colour in African-admixed populations. *Scientific Reports*, *7*(1), 1–9. https://doi.org/10.1038/srep44548

Ji, S. G., Juran, B. D., Mucha, S., Folseraas, T., Jostins, L., Melum, E., Kumasaka, N., Atkinson, E. J., Schlicht, E. M., Liu, J. Z., Shah, T., Gutierrez-Achury, J., Boberg, K. M., Bergquist, A., Vermeire, S., Eksteen, B., Durie, P. R., Farkkila, M., Müller, T., … Anderson, C. A. (2017). Genome-wide association study of primary sclerosing cholangitis identifies new risk loci and quantifies the genetic relationship with inflammatory bowel disease. *Nature Genetics*, *49*(2), 269–273. https://doi.org/10.1038/ng.3745

Kasela, S., Kisand, K., Tserel, L., Kaleviste, E., Remm, A., Fischer, K., Esko, T., Westra, H.-J., Fairfax, B. P., Makino, S., Knight, J. C., Franke, L., Metspalu, A., Peterson, P., & Milani, L. (2017). Pathogenic implications for autoimmune mechanisms derived by comparative eQTL analysis of CD4+ versus CD8+ T cells. *PLOS Genetics*, *13*(3), e1006643. https://doi.org/10.1371/journal.pgen.1006643

Liu, J. Z., Van Sommeren, S., Huang, H., Ng, S. C., Alberts, R., Takahashi, A., Ripke, S., Lee, J. C., Jostins, L., Shah, T., Abedian, S., Cheon, J. H., Cho, J., Daryani, N. E., Franke, L., Fuyuno, Y., Hart, A., Juyal, R. C., Juyal, G., … Weersma, R. K. (2015). Association analyses identify 38 susceptibility loci for inflammatory bowel disease and highlight shared genetic risk across populations. *Nature Genetics*, *47*(9), 979–986. https://doi.org/10.1038/ng.3359

Liyanage, U. E., Law, M. H., Han, X., An, J., Ong, J. S., Gharahkhani, P., Gordon, S., Neale, R. E., Olsen, C. M., MacGregor, S., & Whiteman, D. C. (2019). Combined analysis of keratinocyte cancers identifies novel genome-wide loci. *Human Molecular Genetics*, *28*(18), 3148–3160. https://doi.org/10.1093/hmg/ddz121

López-Isac, E., Acosta-Herrera, M., Kerick, M., Assassi, S., Satpathy, A. T., Granja, J., Mumbach, M. R., Beretta, L., Simeón, C. P., Carreira, P., Ortego-Centeno, N., Castellvi, I., Bossini-Castillo, L., Carmona, F. D., Orozco, G., Hunzelmann, N., Distler, J. H. W., Franke, A., Lunardi, C., … Martin, J. (2019). GWAS for systemic

sclerosis identifies multiple risk loci and highlights fibrotic and vasculopathy pathways. *Nature Communications*, *10*(1). https://doi.org/10.1038/s41467-019-12760-y

Love, M. I., Huber, W., & Anders, S. (2014). Moderated estimation of fold change and dispersion for RNA-seq data with DESeq2. *Genome Biology*, *15*(12), 550. https://doi.org/10.1186/s13059-014-0550-8

Mahajan, A., Taliun, D., Thurner, M., Robertson, N. R., Torres, J. M., Rayner, N. W., Payne, A. J., Steinthorsdottir, V., Scott, R. A., Grarup, N., Cook, J. P., Schmidt, E. M., Wuttke, M., Sarnowski, C., Mägi, R., Nano, J., Gieger, C., Trompet, S., Lecoeur, C., … McCarthy, M. I. (2018). Fine-mapping type 2 diabetes loci to single-variant resolution using high-density imputation and islet-specific epigenome maps. *Nature Genetics*, *50*(11), 1505–1513. https://doi.org/10.1038/s41588-018-0241-6

Martin, M. (2011). Cutadapt removes adapter sequences from high-throughput sequencing reads. *EMBnet.Journal*, *17*(1), 10. https://doi.org/10.14806/ej.17.1.200

Mumbach, M. R., Rubin, A. J., Flynn, R. A., Dai, C., Khavari, P. A., Greenleaf, W. J., & Chang, H. Y. (2016). HiChIP: efficient and sensitive analysis of protein-directed genome architecture. *Nature Methods*, *13*(11), 919–922. https://doi.org/10.1038/nmeth.3999

Okada, Y., Wu, D., Trynka, G., Raj, T., Terao, C., Ikari, K., Kochi, Y., Ohmura, K., Suzuki, A., Yoshida, S., Graham, R. R., Manoharan, A., Ortmann, W., Bhangale, T., Denny, J. C., Carroll, R. J., Eyler, A. E., Greenberg, J. D., Kremer, J. M., … Plenge, R. M. (2014). Genetics of rheumatoid arthritis contributes to biology and drug discovery. *Nature*, *506*(7488), 376–381. https://doi.org/10.1038/nature12873

Paternoster, L., Standl, M., Waage, J., Baurecht, H., Hotze, M., Strachan, D. P., Curtin, J. A., Bønnelykke, K., Tian, C., Takahashi, A., Esparza-Gordillo, J., Alves, A. C., Thyssen, J. P., Den Dekker, H. T., Ferreira, M. A., Altmaier, E., Sleiman, P. M. A., Xiao, F. L., Gonzalez, J. R., … Weidinger, S. (2015). Multi-ancestry genome-wide association study of 21,000 cases and 95,000 controls identifies new risk loci for atopic dermatitis. *Nature Genetics*, *47*(12), 1449–1456. https://doi.org/10.1038/ng.3424

Patro, R., Duggal, G., Love, M. I., Irizarry, R. A., & Kingsford, C. (2017). Salmon provides fast and bias-aware quantification of transcript expression. *Nature Methods*, *14*(4), 417–419. https://doi.org/10.1038/nmeth.4197

Quinlan, A. R., & Hall, I. M. (2010). BEDTools: a flexible suite of utilities for comparing genomic features. *Bioinformatics*, *26*(6), 841–842. https://doi.org/10.1093/bioinformatics/btq033

Ray-Jones, H., Duffus, K., McGovern, A., Martin, P., Shi, C., Hankinson, J., Gough, O., Yarwood, A., Morris, A. P., Adamson, A., Taylor, C., Ding, J., Gaddi, V. P., Fu, Y., Gaffney, P., Orozco, G., Warren, R. B., & Eyre, S. (2020). Mapping DNA interaction landscapes in psoriasis susceptibility loci highlights KLF4 as a target gene in 9q31. *BMC Biology*, *18*(1), 47. https://doi.org/10.1186/s12915-020-00779-3

Schmiedel, B. J., Singh, D., Madrigal, A., Valdovino-Gonzalez, A. G., White, B. M., Zapardiel-Gonzalo, J., Ha, B., Altay, G., Greenbaum, J. A., McVicker, G., Seumois, G., Rao, A., Kronenberg, M., Peters, B., & Vijayanand, P. (2018). Impact of Genetic Polymorphisms on Human Immune Cell Gene Expression. *Cell*, *175*(6), 1701-1715.e16. https://doi.org/10.1016/J.CELL.2018.10.022

Servant, N., Varoquaux, N., Lajoie, B. R., Viara, E., Chen, C.-J., Vert, J.-P., Heard, E., Dekker, J., & Barillot, E. (2015). HiC-Pro: an optimized and flexible pipeline for Hi-C data processing. *Genome Biology*, *16*(1), 259. https://doi.org/10.1186/s13059-015-0831-x

Shi, C., Rattray, M., & Orozco, G. (2019). HiChIP-Peaks: A HiChIP peak calling algorithm. *BioRxiv*, 682781. https://doi.org/10.1101/682781

Smith, T., Heger, A., & Sudbery, I. (2017). UMI-tools: Modeling sequencing errors in Unique Molecular Identifiers to improve quantification accuracy. *Genome Research*, *27*(3), 491–499. https://doi.org/10.1101/gr.209601.116

Stuart, P. E., Nair, R. P., Tsoi, L. C., Tejasvi, T., Das, S., Kang, H. M., Ellinghaus, E., Chandran, V., Callis-Duffin, K., Ike, R., Li, Y., Wen, X., Enerbäck, C., Gudjonsson, J. E., Kõks, S., Kingo, K., Esko, T., Mrowietz, U., Reis, A., … Elder, J. T. (2015). Genome-wide Association Analysis of Psoriatic Arthritis and Cutaneous Psoriasis

Reveals Differences in Their Genetic Architecture. *American Journal of Human Genetics*, *97*(6), 816–836. https://doi.org/10.1016/j.ajhg.2015.10.019

Trynka, G., Hunt, K. A., Bockett, N. A., Romanos, J., Mistry, V., Szperl, A., Bakker, S. F., Bardella, M. T., Bhaw-Rosun, L., Castillejo, G., De La Concha, E. G., De Almeida, R. C., Dias, K. R. M., Van Diemen, C. C., Dubois, P. C. A., Duerr, R. H., Edkins, S., Franke, L., Fransen, K., … Van Heel, D. A. (2011). Dense genotyping identifies and localizes multiple common and rare variant association signals in celiac disease. *Nature Genetics*, *43*(12), 1193–1201. https://doi.org/10.1038/ng.998

Tsoi, L. C., Stuart, P. E., Tian, C., Gudjonsson, J. E., Das, S., Zawistowski, M., Ellinghaus, E., Barker, J. N., Chandran, V., Dand, N., Duffin, K. C., Enerbäck, C., Esko, T., Franke, A., Gladman, D. D., Hoffmann, P., Kingo, K., Kõks, S., Krueger, G. G., … Elder, J. T. (2017). Large scale meta-analysis characterizes genetic architecture for common psoriasis associated variants. *Nature Communications*, *8*(1), 15382. https://doi.org/10.1038/ncomms15382

Võsa, U., Claringbould, A., Westra, H.-J., Bonder, M. J., Deelen, P., Zeng, B., Kirsten, H., Saha, A., Kreuzhuber, R., Kasela, S., Pervjakova, N., Alvaes, I., Fave, M.-J., Agbessi, M., Christiansen, M., Jansen, R., Seppälä, I., Tong, L., Teumer, A., … Franke, L. (2018). Unraveling the polygenic architecture of complex traits using blood eQTL metaanalysis. *BioRxiv*, 447367. https://doi.org/10.1101/447367

Wingett, S., Ewels, P., Furlan-Magaril, M., Nagano, T., Schoenfelder, S., Fraser, P., & Andrews, S. (2015). HiCUP: Pipeline for mapping and processing Hi-C data. *F1000Research*, *4*. https://doi.org/10.12688/f1000research.7334.1

Wishart, D. S., Knox, C., Guo, A. C., Cheng, D., Shrivastava, S., Tzur, D., Gautam, B., & Hassanali, M. (2008). DrugBank: A knowledgebase for drugs, drug actions and drug targets. *Nucleic Acids Research*, *36*(SUPPL. 1), D901. https://doi.org/10.1093/nar/gkm958
